# Targeting TRIM59 impairs RNA splicing and promotes neuroblastoma differentiation and therapeutic responses

**DOI:** 10.1186/s13046-025-03573-7

**Published:** 2025-12-18

**Authors:** Yingwen Zhang, Yi Yang, Guoyu Chen, Minzhi Yin, Yijin Gao, Yanxin Li, Haizhong Feng

**Affiliations:** 1 State Key Laboratory of Systems Medicine for Cancer, Ren Ji Hospital, Shanghai Cancer Institute, Shanghai Jiao Tong University School of Medicine, No. 160 Pu Jian Road, Shanghai, 200127 China; 2 Hainan Academy of Medical Sciences, Hainan Medical University, Haikou, 571199 China; 3 Pediatric Translational Medicine Institute, Department of Hematology & Oncology, National Health Committee Key Laboratory of Pediatric Hematology & Oncology, Shanghai Children’s Medical Center, Shanghai Jiao Tong University School of Medicine, No. 1678, Dong Fang Road, Shanghai, 200127 China; 4 Department of Oncology, Shanghai Children’s Medical Center, Shanghai, 200127 China; 5 Department of Pathology, Shanghai Children’s Medical Center, Shanghai, 200127 China

**Keywords:** TRIM59, Neuroblastoma differentiation, SFPQ, RNA splicing, PRMT1, Chemotherapy, Immunotherapy

## Abstract

**Background:**

Clinical outcomes in neuroblastoma (NB) are closely linked to its differentiation status, making the reversal of differentiation arrest a highly promising therapeutic objective. However, the mechanisms that govern neuronal differentiation in NB remain unclear. In this study, we identify TRIM59 as a key regulator of RNA splicing that drives NB differentiation via an SFPQ-dependent mechanism.

**Methods:**

To identify and target regulators of differentiation in high-risk NB, we collected 98 clinical NB tumor specimens and then performed RNA sequencing (RNA-seq) analysis. The effects of *TRIM59* knockdown on NB differentiation were investigated by immunofluorescence staining, qRT-PCR, western blotting, and IHC staining. The replicate Multivariate Analysis of Transcript Splicing (rMATS) analysis and mass spectrometry was used to assess the role of TRIM59 in RNA splicing and its associated protein, respectively. We developed *TRIM59*-gRNA adeno-associated virus (AAV) to treat animals bearing NB xenograft tumors. The antitumor activity and survival outcomes of AAV-*TRIM59*-gRNA in combination with chemotherapy or CAR-T immunotherapy in vivo were further evaluated.

**Results:**

*TRIM59*-depleted NBs exhibit enhanced neuronal differentiation phenotypes and activation of related signaling pathways, as demonstrated by RNA-seq and functional studies. This effect can be restored by genetically overexpressing the *SEMA4F*-L isoform, but not *SEMA4F*-S, mediated by RNA splicing factor SFPQ. Mechanistically, *TRIM59* deletion promotes differentiation through *SEMA4F*-S isoform upregulation, which is mediated by TRIM59’s control of SFPQ nuclear translocation via the PIN1-importin α axis and PRMT1-dependent asymmetric dimethylation. Therapeutically, adeno-associated virus (AAV)-delivered *TRIM59*-targeting gRNA enhance vincristine (VCR) efficacy by enhancing differentiation. Furthermore, *TRIM59* depletion potentiates B7-H3 CAR-T efficacy via IRF1-L-mediated immunomodulation. Clinical data corroborates TRIM59’s negative correlation with immunotherapy response.

**Conclusions:**

Together, these findings highlight TRIM59’s new functions in RNA splicing and NB differentiation, suggesting that targeting TRIM59 has the potential to improve the efficacy of both chemotherapy and immunotherapy in high-risk NBs.

**Supplementary Information:**

The online version contains supplementary material available at 10.1186/s13046-025-03573-7.

## Background

High-risk neuroblastoma (NB) presents a formidable clinical challenge in children, with an approximate 50% mortality rate [1]. Although significant progress has been made in genomics, proteomics, and single-cell analyses, providing deeper insights into NB biology, these advancements have not yet led to the development of effective therapies [2–5].

Differentiation therapy has become a cornerstone in the treatment of high-risk NBs. Retinoic acids (RA), including all-trans-retinoic acid (ATRA) and 13-cis-retinoic acid (13-cRA), are the most widely utilized drugs in clinical practice to induce differentiation in NB treatment [6, 7]. While RA therapy has been shown to improve survival in some patients, a considerable number, especially those with *MYCN* amplification, eventually develop resistance to the treatment [8, 9]. Currently, the main factors governing NB differentiation remain unidentified.

Alternative splicing (AS) is an evolutionarily conserved mechanism governed by the interplay between splicing factors and cis-regulatory RNA elements [10, 11], which profoundly shapes the diversity and complexity of cancer transcriptomes [12, 13]. Recent studies have highlighted the critical role of RNA splicing factors in tumorigenesis and differentiation of NB [14–17]. *MYCN* amplification, found in nearly half of high-risk NBs, leads to distinct splicing patterns by regulating key factors such as PTBP1 and HNRNPA1 [18, 19]. Moreover, in the TH-*MYCN* NB mouse model, functional mutations within intronic splicing motifs have been linked to tumor progression [17]. These findings suggest that dysregulated mRNA splicing could serve as a valuable therapeutic target in high-risk NBs.

The tripartite-motif (TRIM) family represents a diverse class of RING-type E3 ubiquitin ligases that play critical roles in multiple cellular processes, including cell proliferation, differentiation, immune response, and tumorigenesis [20–22]. Recent studies have underscored the critical role of TRIM proteins in promoting stem cell characteristics and maintaining a stem-like phenotype in cancer cells through distinct molecular pathways [23–26]. TRIM59, a core member of the TRIM family, plays a pivotal role in regulating embryonic differentiation and maintaining tumor stemness [27–29]. In NBs, TRIM59 has been shown to regulate tumorigenicity via the Wnt/β-catenin and p53 signaling pathways [30, 31]. However, its precise role in NB differentiation remains poorly understood.

In this study, we report a crosstalk between RNA splicing and NB differentiation, which is linked by TRIM59. TRIM59 modulates SFPQ-mediated RNA AS events by influencing the nuclear translocation of SFPQ and enhancing its asymmetric dimethylarginine modification by PRMT1, which thus confers poorly tumor differentiated states and therapeutic resistance. Treatment with adeno-associated virus (AAV)-mediated *TRIM59* gRNA, alone or combined with vincristine (VCR) or B7-H3 CAR-T cell therapy, significantly reduces NB burden, suggesting that targeting TRIM59 represents a potential therapeutic strategy to enhance the efficacy of chemotherapy and immunotherapy in high-risk NBs.

## Methods

### Clinical samples

All clinical NB patient specimens were collected from Shanghai Children’s Medical Center (SCMC). The experimental procedures were approved by the ethics committee of Shanghai Children’s Medical Center, School of Medicine, Shanghai Jiao Tong University, Shanghai, China (SCMCIRB-K2023093-1). Written informed consent was obtained from all participants before surgery. These specimens were examined and diagnosed by pathologists at SCMC.

### Cell line and culture

The SK-N-BE(2) and HEK293 cell lines were obtained from ATCC (Manassas, VA, USA, CRL-2271; CRL-3216) and cultured in DMEM supplemented with 10% fetal bovine serum (FBS) and 1% penicillin-streptomycin (PS). The CHP-134 cell line was obtained from DSME (Braunschweig, Germany, ACC-653) and cultured in RPMI 1640 supplemented with 10% FBS and 1% PS. All cell lines were cultured at 37 °C in a humidified 5% CO_2_ incubator. All cell lines were recently authenticated using STR DNA fingerprinting at Shanghai Biowing Applied Biotechnology Co., Ltd (Shanghai, China), and mycoplasma infection was detected using LookOut Mycoplasma PCR Detection Kit (Sigma-Aldrich).

### Antibody

The following primary antibodies were used in this study: GAPDH (1:1000 for WB, 10494-1-AP, Proteintech), TRIM59 (1:500 for WB, 1:500 for IF, 1:1200 for IHC, ab69639, Abcam), SFPQ (1:2000 for WB, 1:200 for IF, SAB4200501, Sigma), SOX2 (1:1000 for WB, ab92494, Abcam), TUJ1 (1:1000 for WB, 1:1000 for IF, 801213, Biolegend), MYCN (1:1000, 84406, Cell Signaling Technology), Ki67 (1:500, 9449, Cell Signaling Technology), MAP2 (1:250 for IHC, 4542, Cell Signaling Technology), Lamin B1 (1:5000, 66095-1-Ig, Proteintech), PIN1 (1:1000 for WB, sc-46660, Santa Cruz), pan-Asymmetric-Di-Methyl Arginine (1:1000 for WB, M005375, Abmart), HA-Tag (1:5000 for WB, 51064-2-AP, Proteintech), PRMT1 (1:1000 for WB, 2449, CST), Flag-Tag (1:3000 for WB, 66008-4-Ig, Proteintech), Myc-Tag (1:1000 for WB, M20002, Abmart), NDRG1 (1:1000 for WB, 26902-4-Ig, Proteintech).

### Plasmids

The shRNA oligonucleotides targeting *TRIM59*, *SFPQ*, *PIN1*, and *PRMT1* were cloned into pLKO.1-Puro vectors. Plasmids containing HA-tagged *TRIM59*, *TRIM59*-truncated constructs, Flag-tagged importin α5, and Flag-tagged *PIN1* have been described previously ^[32]^. The pLV3-HA-*SFPQ* and pCMV-Myc-*PRMT1* plasmids were purchased from MiaoLing Biology (WuHan, China). *SFPQ*-truncated constructs were cloned into pLV3-HA-Puro vectors. *SFPQ* methylation-deficient mutations were generated by a Site-Directed Mutagenesis Kit (Invitrogen) according to the manufacturer’s instructions. *SEMA4F*-S, *SEMA4F*-L, *IRF1*-S and *IRF1*-L were PCR-amplified from cDNAs of SK-N-BE(2) cells and subsequently cloned into pLenti-CMV-blast vectors.

### RNA purification and qRT-PCR

Total RNA was purified using the TRIzol Plus RNA Purification Kit (12183555, Thermo Fisher), and was reverse-transcribed into cDNA with the High Capacity cDNA Reverse Transcription Kit (4368813, Thermo Fisher). qRT-PCR was performed using the SYBR Green PCR Kit (Life Technologies) on a CFX Connect Real-Time System (BIO-RAD). Primers used are listed in Extended Data Table 5. Results were analyzed using the 2^−(ΔΔCt)^ method.

### RNA-seq and alternative splicing analysis

RNA-seq was performed as described previously [32]. RNA quality was assessed using a Bioanalyzer before sequencing. RNA libraries were constructed using the Illumina TruSeq RNA library preparation kit and sequenced on the Novaseq S4 PE150 platform. Alternative splicing (AS) events were identified using an rMATS analysis, which classified the events into five categories, including MXE, A3SS, A5SS, SE, and RI.

### Cell proliferation and colony formation assays

Cell proliferation was analyzed with Cell Counting Kit-8 (CCK8) (40203ES60, Yeasen) according to the manufacturer’s instructions. For the colony formation assays, 2000–4000 cells were seeded in a six-well plate for 14 days, and the cell culture medium was changed every three days. The colonies were stained with crystal violet and counted after imaging.

### Immunofluorescence (IF) analysis

Cells grown on coverslips were fixed with 4% paraformaldehyde for 15 min, permeabilized with 0.2% Triton X-100 in PBS for 10 min, and then blocked with 10% bovine serum albumin in PBS for 1 h at room temperature. The cells were incubated with the primary antibody at 4 °C overnight. On the following day, the cells were washed twice with PBS and incubated with the secondary antibodies for 1 h. Nuclei were stained with 4‘,6-diamidino-2-phenylindole (DAPI). Samples were analyzed using a Leica SP8 confocal laser scanning microscope.

### Proximity ligation assay (PLA)

PLAs were performed using a Duolink PLA Starter Kit (Sigma), following the manufacturer’s instructions. Briefly, cells grown on coverslips were fixed in 4% paraformaldehyde for 15 min, permeabilized with 0.2% Triton X-100 in PBS for 10 min, and blocked with provided blocking buffer at 37 °C for 1 h. The cells were incubated overnight at 4 °C with the primary antibody.

On the following day, the sections were incubated sequentially with PLA probes (37 °C for 1 h), ligation solution (37 °C for 30 min), and amplification solution (37 °C for 100 min). Afterward, nuclei were counterstained with DAPI. Samples were analyzed using a Leica SP8 confocal laser scanning microscope.

### Immunoprecipitation (IP) and Western blotting analyses

Cells were lysed in IP lysis buffer (20 mM Tris-HCl, pH 7.4, 150 mM NaCl, 0.5% Triton X-100, 0.5% deoxycholate, 1 mM DTT) supplemented with protease inhibitors at 4 °C for 10 min. The cell lysates were centrifuged at maximum speed for 15 min. Equal amounts of supernatant were incubated with protein A/G-agarose beads (Invitrogen) and the indicated antibodies at 4 °C overnight. The precipitated beads were washed three times with bead wash buffer (20 mM Tris-HCl, pH 7.4, 150 mM NaCl, 0.5% Triton X-100, 1 mM EDTA) and eluted in 40 µL 2× SDS-PAGE loading buffer by boiling for 10 min. For immunoblotting, equal protein amounts from input and IP fractions were resolved by SDS-PAGE and transferred to NC membranes. Specifically, 5–10% of the input fraction and 10 µL of immunoprecipitate were analyzed. Western blotting was performed using the indicated primary antibodies.

### RNA Immunoprecipitation

About 2 × 10^7^ cells were washed with PBS three times and lysed in 100 µL RIP lysis buffer (0.1 mM KCl, 5 mM MgCl_2_, 10 mM HEPES-NaOH, pH 7, 0.5% NP-40) supplemented with proteinase inhibitor, 1 mM DTT and 200 U/mL RNase inhibitor at 4 °C for 10 min. The cell lysates were then centrifuged at maximum speed for 15 min at 4 °C. At the same time, magnetic beads were incubated with indicated antibodies for 1 h at room temperature. Next, 100 µL of lysate supernatant was mixed with bead-antibody complex and 900 µL RIP immunoprecipitation buffer (50 mM Tris-HCl, pH 7.4, 150 mM NaCl, 1 mM MgCl_2_, 0.05% NP-40, 20 mM EDTA, pH 8.0, 1 mM DTT, 200 U/mL RNase inhibitor) and incubated at 4 °C overnight. The precipitated beads were washed five times with RIP wash buffer (50 mM Tris-HCl, pH 7.4, 150 mM NaCl, 1 mM MgCl_2_, 0.05% NP-40) and resuspended in 150 µL Proteinase K buffer (126 µL RIP wash buffer, 15 µL 10% SDS, 9 µL 20 mg/mL proteinase K) at 55 °C for 30 min. After the incubation, the supernatant was transferred to a new tube and 1 mL of TRIzol was added. Then, the bound RNA was detected by qRT-PCR. Primers used were listed in Supplemental Table 5.

### Cell viability assay

The SK-N-BE(2) or CHP-134 cells were seeded into 96-well plates at a density of 5 × 10^3^ cells per well. The cells were treated with different doses of VCR, olaparib,13-cRA, CTX, or VP16. Cell viability assays were performed with CellTiter-Glo^®^ Luminescent Cell Viability Assay Kit (Promega) according to the manufacturer’s instructions.

### Apoptosis detection

Cells were harvested and washed twice with cold PBS. Following this, the cells were resuspended in 250 µL of 1× binding buffer. Apoptosis was assessed using the Annexin V-PE/7-AAD Apoptosis Detection Kit (40310ES50, Yeasen) according to the manufacturer’s instructions, and subsequently analyzed with a flow cytometer.

### Flow cytometry

Cells were incubated with specific surface antibodies or isotype controls for 30 min on ice. For intracellular IFN-γ^+^ analysis, GolgiStop and GolgiPlug (BD Biosciences) were added to the culture 4 h prior to detection, following the manufacturer’s instructions. Cells were subsequently fixed and permeabilized using the BD Cytofix/Cytoperm™ Plus Fixation/Permeabilization Kit and stained with anti-IFN-γ BV421 antibody. After staining, cells were washed three times with PBS and analyzed using a BD FACSCalibur flow cytometer. Data were processed with FlowJo software (v10.9.0). Anti-CD3 (PerCP/Cyanine5.5, 317335) and anti-IFN-γ (BV421, 506537) antibodies were purchased from BioLegend.

### Cytotoxicity assay

CD19 CAR-T and B7-H3 CAR-T cells were generated as previously reported, which were gifts from Dr. Li [33]. CD19 CAR-T cells were used as a negative control. CAR-T cells and NB tumor cells were co-cultured at effector-to-target (E: T) ratios of 1:3 or 1:4. Cells were analyzed to measure residual tumor cells and T cells by FACS at various time points (48, 72, 96, and 120 h).

### AAV virus production

As previously report [34], a *TRIM59* gRNA was inserted into the pAAV-CMV-SauriCas9 vector. The sgRNA targeting *TRIM59* was designed using CRISPR RGEN Tools (http://www.rgenome.net/*)*, and primers used are listed in Supplemental Table 5. The pAAV-CMV-SauriCas9-TRIM59-sgRNA, pRC2-mi342, and pHelper (TAKARA, 6234) were co-transfected into AAVpro 293 T cells (TAKARA, 632273) using PEI MAX (Polysciences, 24765). At 72 h post-transfection, the culture supernatant and cell pellet were collected. AAV virus extraction (TAKARA, 6235), concentration (TAKARA, 6674), purification (TAKARA, 6675), and titer determination (TAKARA, 6233) were performed according to the manufacturer’s instructions.

### Animal experiments

All animal experiments were conducted under the Institutional Animal Care and Use Committee (IACUC)-approved protocols at SCMC following NIH and institutional guidelines. The approval number was SCMC-LAWEC-2023-061. Tumor volumes were measured every other day, using the formula: length × width² × 0.5. The maximum tumor size did not exceed 1500 mm³.

### Mouse xenograft tumor model

CB17-SCID female mice aged 6–8 weeks (SLAC, Shanghai, China) were used. Mice were randomly divided into five per group. A total of 5 × 10⁶ NB cells, transfected with either the vehicle or the indicated vector, were subcutaneously injected into animals. Tumor growth was monitored, and when tumors in the control group reached an average size of 1000–1500 mm³, mice were euthanized. Tumors were then excised for subsequent histological analysis.

### Adeno-associated virus treatments

5 × 10^6^ NB cells were subcutaneously injected into NSG mice. On day 7 post-tumor implantation, the mice were randomized and treated with intratumoral injections of either AAV-*TRIM59* sgRNA or AAV- control sgRNA. In the mouse model combining AAV treatment and chemotherapy, mice received a 3-week course of intraperitoneal vincristine (VCR; 0.5 mg/kg, five days per week) or control injections, starting on day 14 post-transplantation. In the mouse model combining AAV treatment and immunotherapy, 2 × 10^6^ CD19 or B7-H3 CAR-T cells were administered intravenously on day 14 and 21 post-tumor implantation.

### Immunohistochemistry

Paraffin sections were dewaxed, rehydrated, and subjected to antigen retrieval using 0.01 M sodium citrate buffer. Subsequently, the sections were treated with 3% hydrogen peroxide to inactivate endogenous peroxidase and blocked with 10% goat serum in PBS. Then, the sections were incubated with primary antibodies overnight at 4 °C. The following day, sections were incubated with secondary antibodies and developed using a DAB kit (GK600710, GT). Nuclei were counterstained with hematoxylin. Normal rabbit IgG was used as a negative control. The proportion of positive cells was quantified using Image-Pro Plus 6.0.

### Statistical analysis

Statistical analyses were performed using GraphPad Prism 8.0 for Windows (GraphPad Software Inc., San Diego, CA, USA). An unpaired, two-tailed Student’s *t*-test and one-way ANOVA were utilized to assess differences between groups. Kaplan-Meier survival analysis was conducted using log-rank tests. Correlation analysis was assessed by the Pearson method. A *P*-value of < 0.05 was considered statistically significant.

## Results

### Elevated TRIM59 expression correlates with undifferentiated state, chemoresistance, and T cell exclusion in NB

**Fig. 1:**
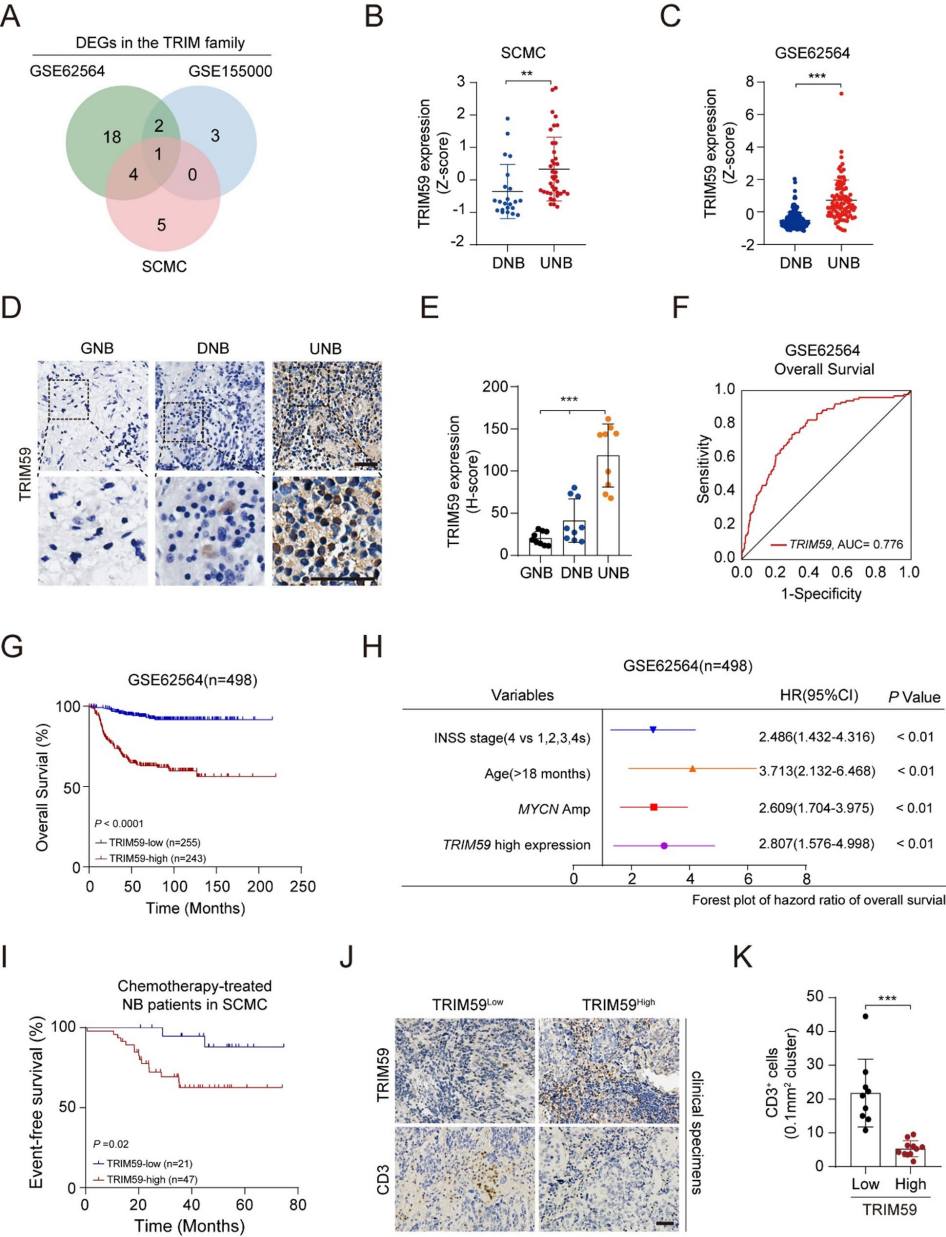
TRIM59 is an independent NB prognostic factor, with elevated expression correlating with undifferentiated histology, chemoresistance, and T cell exclusion. **A** Venn diagram showing the overlap of *TRIM59* expression between undifferentiated and differentiated NB samples from the SCMC and GSE62564 datasets, with downregulation following ATRA-induced differentiation in *MYCN*-amplified NB cell lines from the GSE155000 dataset. Statistical significance was determined with *P* < 0.05. **B** *TRIM59* expression in differentiated (DNB, *n* = 22) and undifferentiated (UNB, *n* = 39) NB cases from the SCMC dataset. **C** *TRIM59* expression in differentiated (DNB, *n* = 181) and undifferentiated (UNB, *n* = 91) NB samples from the GSE62564 dataset. **D **TRIM59 protein expression levels in ganglioneuroblastomas (GNB), differentiated (DNB), and undifferentiated NBs (UNB). Scale bar, 50 μm. **E** Quantification of TRIM59-positive cells from (D). *** *P* < 0.001, by one-way ANOVA. **F** Receiver operating characteristic (ROC) curve analysis of *TRIM59* expression’s prognostic potential in NB from the GSE62564 dataset. **G** Kaplan-Meier survival curves for overall survival in the GSE62564 dataset, stratified by *TRIM59* expression (low vs. high), with the optimal cut-off value (12.472) determined by the R package. Statistical significance was assessed using the log-rank test. **H** Cox regression analysis of the prognostic ability of *TRIM59* expression in NB. **I **Event-free survival analysis of chemotherapy-treated NB patients from the SCMC dataset, stratified by *TRIM59* expression (high vs. low), with the optimal cut-off value (46.372) determined by the R package. Statistical significance was assessed using the log-rank test. **J** CD3 expression in NB patients stratified by high or low TRIM59 expression levels. Scale bar, 50 μm Quantification of CD3-positive cells in samples from (J). Data represent three independent experiments. Error bars indicate mean ± s.d. *P < 0.05, ** *P* < 0.01, *** *P* < 0.001, by two-tailed *t*-test

To discover actionable regulators of NB differentiation, we integrated RNA-sequencing data from Shanghai Children’s Medical Center (SCMC, Supplemental Table 1) with the publicly available GSE62564 dataset. Differential expression analysis of TRIM family genes revealed significant downregulation of *TRIM54*, *TRIM59*, *TRIM28*, *TRIM71*, and *TRIM24* in differentiated tumors (Fig. 1, A-C, and Supplemental Fig. 1, A and B). Notably, *TRIM59* was uniquely and consistently downregulated upon ATRA-induced differentiation in *MYCN*-amplified BE-C and NGP cell lines (Fig. 1A, and Supplemental Fig. 1 C). Clinical validation showed reduced TRIM59 protein in clinical ganglioneuroblastomas (GNBs) and differentiated NBs compared to undifferentiated specimens (Fig. 1, D and E). Survival analysis based on the GSE62564 cohort established a significant association between *TRIM59* expression levels and clinical prognosis in NB patients (Fig. 1F). Importantly, elevated *TRIM59* expression correlated with poor prognosis, showing a significant association with reduced overall survival (Fig. 1, F and G) and severing as an independent prognostic biomarker (Fig. 1H). In all, these results establish TRIM59 as both a key maintainer of the undifferentiated state and a powerful predictor of clinical outcomes in NB.

High-risk NB exhibits poor responsiveness to differentiation therapy and frequently develops therapeutic resistance [35]. RA-based differentiation therapy combined with surgery and chemotherapy has been shown to improve outcomes, particularly through the administration of 13-cRA following chemotherapy, which significantly prolongs event-free survival [36, 37]. To investigate the role of TRIM59 in treatment sensitivity, we assessed the influence of its expression on chemosensitivity in high-risk NB. Notably, patients with low *TRIM59* expression in NBs exhibited better responses to chemotherapy and had longer event-free survival, as observed in the SCMC dataset (Fig. 1I, and Supplemental Table 1), suggesting that TRIM59 may contribute to chemotherapy resistance. Furthermore, given the established benefit of combining immunotherapy with ATRA-based post-consolidation therapy in high-risk NB [38, 39], we assessed TRIM59’s role in immune infiltration. As shown in Fig. 1J and K, clinical NBs with low TRIM59 protein expression exhibited higher CD3^+^ T cell infiltration compared to those with high TRIM59 expression. These results suggest that TRIM59 expression is associated with chemotherapy resistance and reduced T cell infiltration in NB, indicating a potential link between TRIM59 expression and the tumor immune microenvironment, and highlighting its potential relevance to therapeutic responses.

### Inhibition of TRIM59 promotes NB tumor differentiation towards mature neuroblasts

**Fig. 2:**
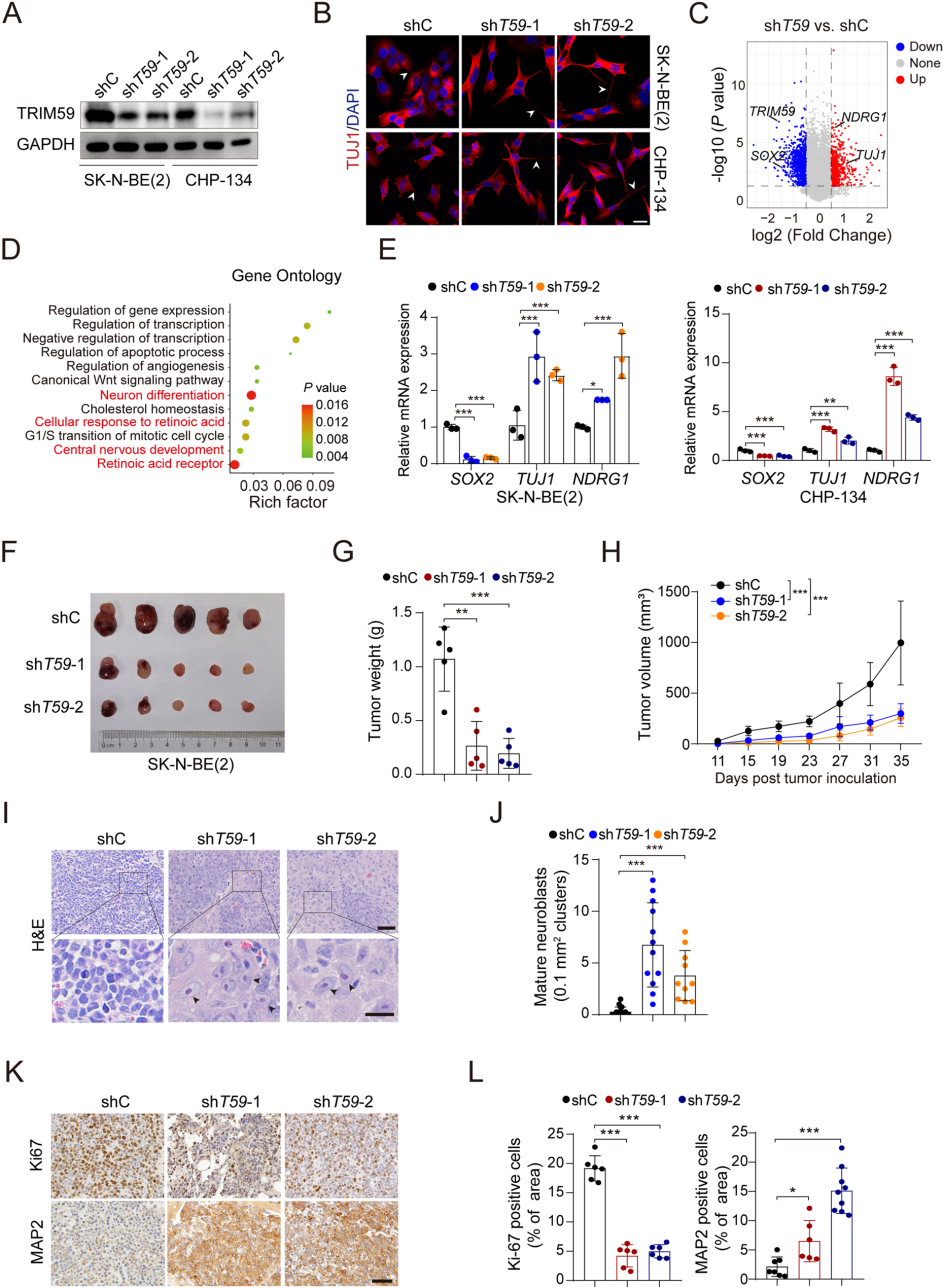
Inhibition of TRIM59 promotes NB tumor differentiation towards mature neuroblasts. **A **WB analysis showing *TRIM59* knockdown (KD) in SK-N-BE(2) and CHP-134 cells. **B** Representative images of immunofluorescence (IF) staining in SK-N-BE(2) and CHP-134 cells transfected with control shRNA (shC) or *TRIM59* shRNAs (shT59-1 or −2). TUJ1 is shown in red, and DAPI in blue. Scale bar, 10 μm. **C** Volcano plot illustrating differentially expressed genes (DEGs) between control and *TRIM59* KD SK-N-BE(2) cells, with |log2FC| > 0.5, *P* < 0.05. **D** Gene Ontology (GO) enrichment analysis of DEGs following *TRIM59* KD in SK-N-BE(2) cells. **E** Relative mRNA expression levels of *SOX2*, *TUJ1* and *NDRG1. ***F **Representative images of tumors from 6-week-old CB17-SCID mice injected with SK-N-BE(2) cells transfected with shC or sh*TRIM59* at 35 days. **G** Quantification of tumor weight from (F). **H** Tumor growth curves measured in xenograft mice. **I **Hematoxylin and eosin (H&E) staining of xenograft tumor sections. Top panel: Scale bar, 50 μm. Bottom panel: Scale bar, 20 μm. **J** Quantification of mature neuroblasts in xenograft tumor sections. **K** IHC staining for Ki67 and MAP2 in the xenograft tumor sections. Scale bar, 50 μm. **L** Quantification of Ki67- and MAP2-positive cells in (K). Error bars indicate mean ± s.d. *P < 0.05, ** *P* < 0.01, *** *P* < 0.001, by two-tailed *t*-test

To investigate whether TRIM59 is required for NB differentiation, we performed *TRIM59* knockdown (KD) in two *MYCN*-amplified human NB cell lines, SK-N-BE(2) and CHP-134, using two distinct small hairpin RNAs (shRNAs; sh*T59*−1 and sh*T59*−2) or a control shRNA (shC; Fig. 2A). Moreover, *TRIM59* KD induced pronounced morphological changes, including neurite outgrowth, a hallmark of NB differentiation (Supplemental Fig. 1D). *TRIM59* KD enhanced neuronal differentiation was further supported by immunofluorescence staining of TUJ1, a marker for differentiated neurons, and quantitative analysis of neurite lengths (Fig. 2B, and Supplemental Fig. 1E). To explore the underlying mechanisms of NB differentiation by *TRIM59* KD, we performed RNA sequencing (RNA-seq) in SK-N-BE(2) cells (Fig. 2C). *TRIM59* KD cells were enriched in genes associated with neuronal differentiation and retinoic acid receptor signaling pathways (Fig. 2D). We further analyzed the expression of neuronal differentiation markers and found that *TRIM59* depletion led to increased expression of differentiation markers *NDRG1* and *TUJ1*, along with a marked reduction in the pluripotency marker *SOX2* in both SK-N-BE(2) and CHP-134 cells (Fig. 2E). Additionally, the protein levels of TUJ1 and NDRG1 were elevated, while SOX2 protein expression was notably reduced following *TRIM59* KD (Supplemental Fig. 1F). As previously reported [31], *TRIM59* KD suppressed cell proliferation (Supplemental Fig. 1, G-I). These findings indicate that *TRIM59* depletion enhances neuronal differentiation in NB cells, supporting a potential role of TRIM59 in the regulation of NB differentiation.

To further evaluate the role of TRIM59 in NB progression in vivo, we injected *TRIM59* KD SK-N-BE(2) cells and their corresponding control cells into the subcutaneous tissues of immunodeficiency mice to induce NBs. *TRIM59* KD markedly reduced tumor growth (Fig. 2, F-H). Notably, the *TRIM59* KD group displayed marked changes in cell morphology, resembling the pathological features of mature neuroblasts (Fig. 2, I and J). The expression levels of Ki67, a marker of aggressive proliferation, were significantly reduced in the *TRIM59* KD group, whereas MAP2, a marker of neuronal dendrites, exhibited a marked increase (Fig. 2, K and L), further supporting the role of TRIM59 in the control of NB differentiation.

### TRIM59 interacts with RNA splicing factor SFPQ

**Fig. 3:**
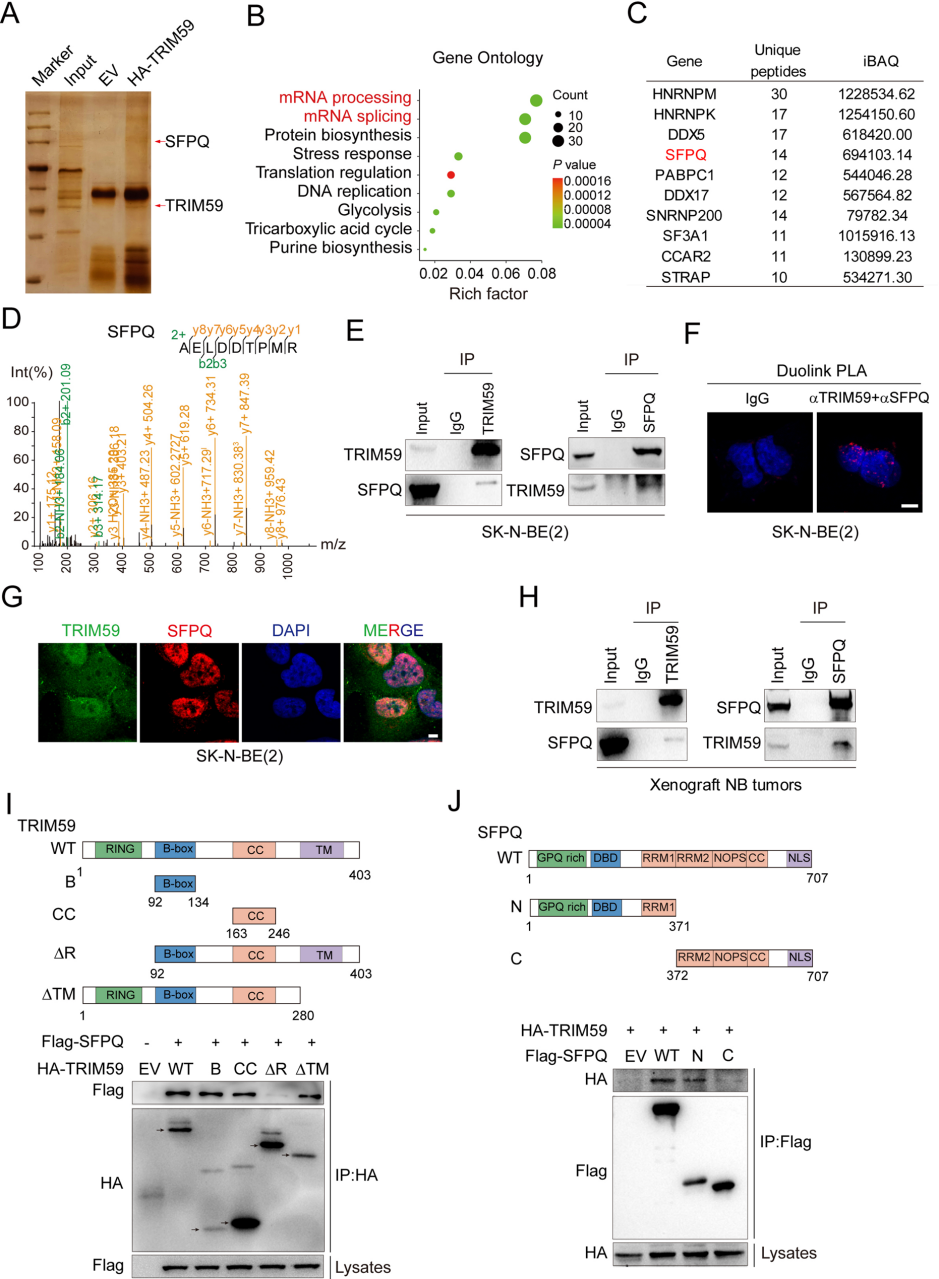
TRIM59 interacts with SFPQ. **A** IP assay with an anti-HA antibody, followed by liquid chromatography-mass spectrometry (LC-MS) analysis. SK-N-BE(2) cells stably expressed HA-TRIM59 or an empty vector (EV). **B **Gene Ontology (GO) analysis identifying functional categories associated with TRIM59-interacting partners. **C** The top 10 TRIM59-binding RNA splicing regulators ranked by the number of unique peptides identified. **D** Mass spectrometry (MS) identified SFPQ peptides among TRIM59-interacting proteins. **E** IP and WB of the association between TRIM59 and SFPQ in SK-N-BE(2) cells. **F** Representative PLA images showing interactions between TRIM59 and SFPQ in SK-N-BE(2) cells. Scale bar, 5 μm. **G** IF staining of TRIM59 (green), SFPQ (red), and DAPI (blue) in SK-N-BE(2) cells. Scale bar, 10 μm. **H** IP and WB of the association between TRIM59 and SFPQ in xenograft NB tumors. **I** Co-IP of Flag-tagged SFPQ with either full-length or truncated mutants of HA-tagged TRIM59. Arrow, target protein. **J** Co-IP of HA-tagged TRIM59 with either full-length or truncated mutants of Flag-tagged SFPQ

To elucidate the mechanism of TRIM59 in regulating NB differentiation, we performed mass spectrometry on SK-N-BE(2) cells to identify TRIM59 cofactors (Fig. 3A). Compared to the empty vector (EV), 481 proteins were identified to specifically bind to HA-tagged TRIM59, based on a screening criterion of at least six unique peptides (Supplemental Table 2). These proteins were predominantly enriched in mRNA processing and splicing pathways (Fig. 3B), suggesting a role for TRIM59 in RNA splicing. SFPQ (splicing factor proline- and glutamine-rich) was ranked among the top 10 RNA splicing regulators interacting with TRIM59 (Fig. 3, C and D). Their interaction was confirmed by immunoprecipitation assays (Fig. 3E). Proximity ligation assay (PLA) revealed in situ TRIM59-SFPQ complexes localized at discrete nuclear foci (Fig. 3F). The nuclear co-localization of TRIM59 and SFPQ was additionally validated by immunofluorescence staining (Fig. 3G). Notably, TRIM59 was also detected in the cytoplasm, consistent with findings from our group and others [40–42]. Furthermore, the interaction between TRIM59 and SFPQ was confirmed in xenograft NB tumors (Fig. 3H).

To identify the domains mediating the interaction between TRIM59 and SFPQ, we generated HA-tagged TRIM59 and Flag-tagged SFPQ constructs. As shown in Fig. 3I and J, the B-box, CC and RING domains were required for their interaction. In parallel, SFPQ-N (1–371 amino acid residues), but not SFPQ-C, successfully pulled down full-length TRIM59, indicating that the N-terminal region of SFPQ is essential for their binding. These data collectively indicate that TRIM59 associates with SFPQ, likely through the B-box, CC and RING domains of TRIM59 and the N-terminal region of SFPQ.

### TRIM59 regulates RNA alternative splicing through SFPQ

**Fig. 4:**
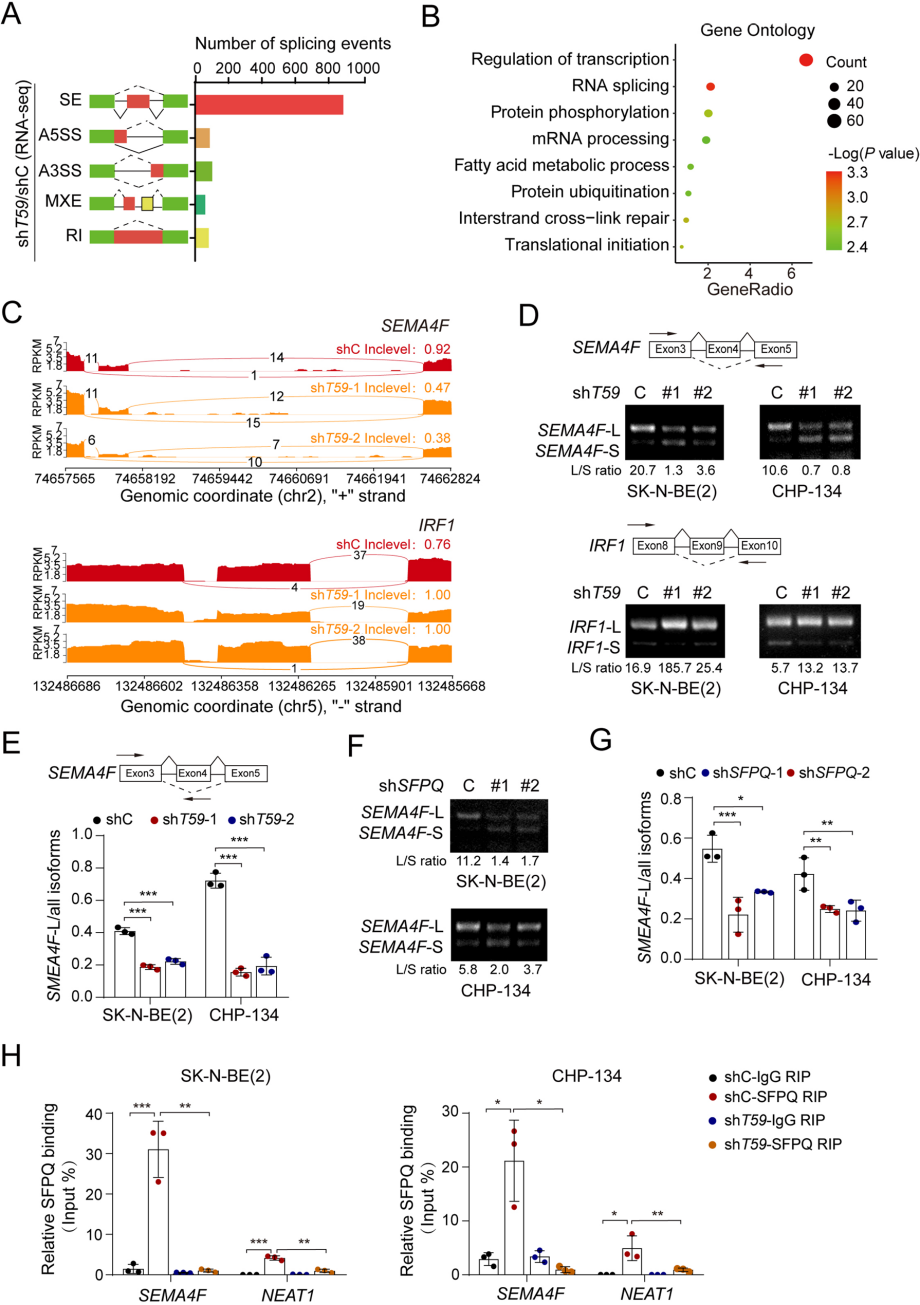
TRIM59 regulates RNA alternative splicing through SFPQ. ** A **Quantification of AS events affected by *TRIM59* KD. Events are categorized into SE, A5SS, A3SS, MXE, and RI. **B **GO analysis of TRIM59-mediated AS target genes. **C **Sashimi plots depicting the splicing patterns of *SEMA4F* and *IRF1* transcripts in SK-N-BE(2) cells transfected with shC or sh*TRIM59.* **D **Schematic of the strategy to identify splicing events in *SEMA4F* and *IRF1*. Splicing events were verified by RT-PCR in SK-N-BE(2) and CHP-134 cells. **E **qRT-PCR of the *SEMA4F*-L to total *SEMA4F* isoforms ratio. **F** RT-PCR of *SEMA4F* splicing events. **G** qRT-PCR of the *SEMA4F*-L to total *SEMA4F* isoforms ratio. **H** RNA immunoprecipitation (RIP) assays demonstrating the association of SFPQ with *SEMA4F* pre-mRNAs, which was disrupted following *TRIM59* depletion. IgG, an isotype control. *NEAT1*, a positive control. Data represent three independent experiments. Error bars indicate mean ± s.d. ** *P* < 0.01, *** *P* < 0.001, by two-tailed *t*-test

To investigate the role of TRIM59 in regulating RNA alternative splicing events, we conducted rMATS (replicate Multivariate Analysis of Transcript Splicing) analysis on RNA sequencing (RNA-seq) data from SK-N-BE(2) cells stably transduced with either a control shRNA (shC) or two different *TRIM59* shRNAs (Supplemental Table 3). The screening criteria were set to an *FDR* (false discovery rate) < 0.05. *TRIM59* KD resulted in more than 1,000 AS events shared by two distinct *TRIM59*-targeted shRNAs, including 893 skipped exons (SE), 93 alternative 5’ splice sites (A5SS), 108 alternative 3’ splice sites (A3SS), 66 mutually exclusive exons (MXE), and 87 retained introns (RI) (Fig. 4A). Given that the SE accounted for the majority of alternative splicing events, we hypothesized that SE represents the primary mode of transcriptional regulation mediated by TRIM59. We then performed Gene Ontology (GO) term analysis and revealed that TRIM59-influenced genes were closely associated with RNA transcription, RNA splicing, and mRNA processing (Fig. 4B), suggesting a close association between TRIM59 and mRNA splicing. Additionally, following *TRIM59* KD, a comparable proportion of exon inclusion and exon skipping was observed (Supplemental Fig. 2 A), indicating a dual functionality of TRIM59 as both a splicing activator and repressor.

Among all TRIM59-affected SE events, 98 genes exhibited significant alterations (Supplemental Table 4). Differentiation-related genes among these were selected for further validation by RT-PCR. As shown in Fig. 4C and D, *TRIM59* KD significantly impaired *SEMA4F* and *IRF1* splicing. Since the regulation of *IRF1* splicing by SFPQ was consistent with a previous report [43], *SEMA4F* was chosen for further investigation. *TRIM59* KD decreased the relative proportion of the *SEMA4F*-L isoform within the total *SEMA4F* mRNA pool by qRT-PCR analysis (Fig. 4E).

To investigate whether TRIM59 regulates AS events through SFPQ, we knocked down *SFPQ* using two distinct shRNAs in SK-N-BE(2) and CHP-134 cells (Supplemental Fig. 2B). *SFPQ* KD did not alter TRIM59 expression (Supplemental Fig. 2B) but led to a marked reduction in cell proliferation (Supplemental Fig. 2C). Importantly, depletion of *SFPQ* markedly promoted cellular morphological differentiation (Supplemental Fig. 2, D and E) and upregulated the expression of differentiation-related markers (Supplemental Fig. 2, F and G). Consistent with the changes observed in *TRIM59* KD cells, the levels of *SEMA4F*-L isoform significantly decreased following SFPQ inhibition (Fig. 4, F and G), suggesting that SFPQ acts as an intermediate in TRIM59-regulated *SEMA4F* splicing. Like the positive control gene, long non-coding RNA (lncRNA) NEAT1 [44], *SEMA4F* pre-mRNAs were effectively enriched by SFPQ in both cell lines (Fig. 4H). Moreover, *TRIM59* KD significantly reduced the binding of SFPQ to *SEMA4F* pre-mRNAs (Fig. 4H), indicating that TRIM59 regulates RNA splicing through SFPQ.

### TRIM59 promotes nuclear translocation of SFPQ through PIN1/Import α axis

**Fig. 5:**
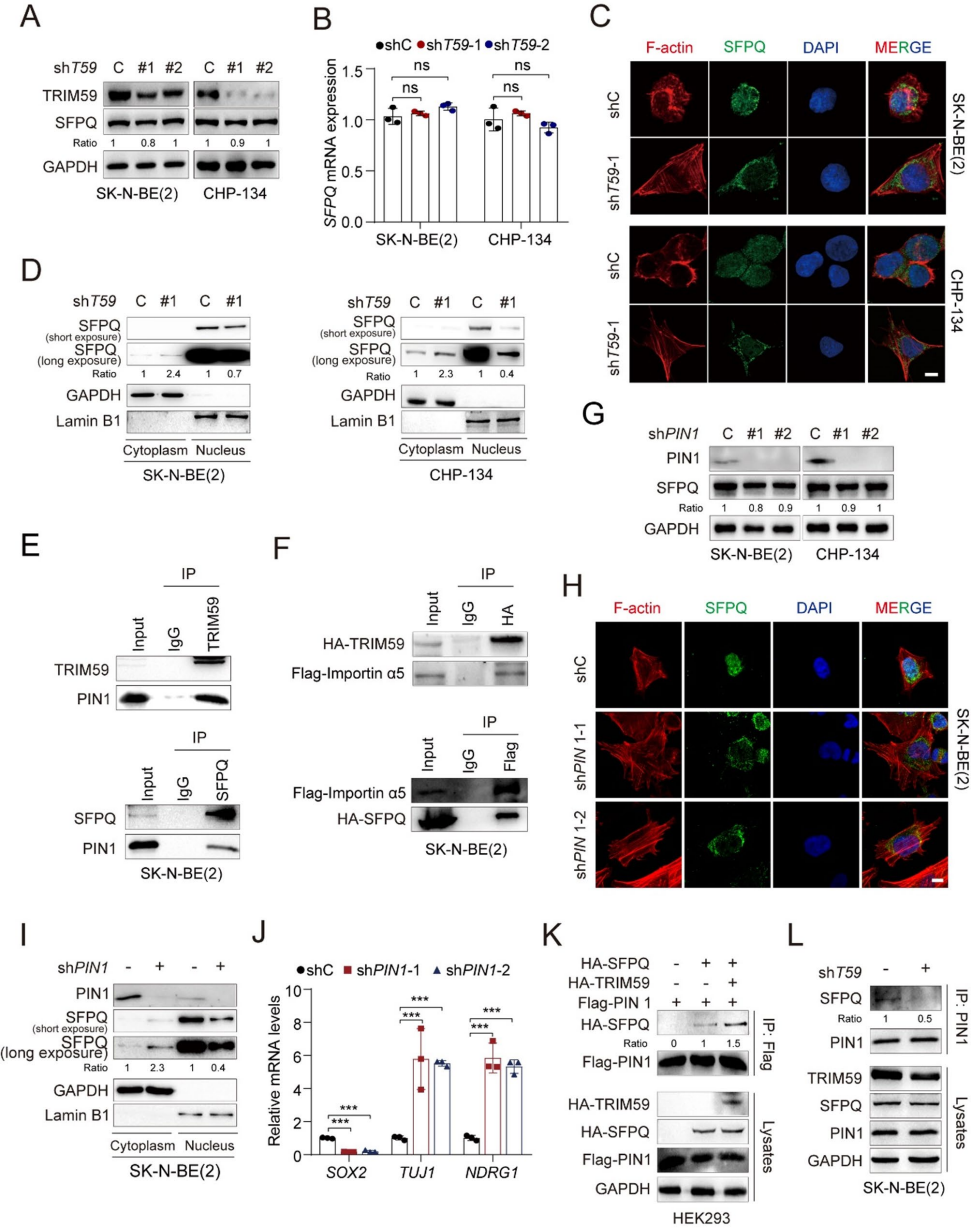
TRIM59 promotes SFPQ nuclear translocation through PIN1/Importin α. **A **Effect of *TRIM59* depletion on SFPQ protein expression in CHP-134 and SK-N-BE(2) cells. **B **Effect of *TRIM59* depletion on *SFPQ* mRNA expression. ns, not significant. **C** IF of F-actin (red), SFPQ (green), and DAPI (blue) in CHP-134 and SK-N-BE(2) cells transfected with shC or sh*TRIM59*. *TRIM59* depletion resulted in decreased SFPQ nuclear localization and increased its cytosolic localization. Scale bar, 10 μm. **D** WB of the effect of *TRIM59* depletion on SFPQ cellular localization. **E** IP of the interaction among TRIM59, SFPQ, and PIN1 in SK-N-BE(2) cells. **F** IP of the interaction among TRIM59, SFPQ, and Import α5 in SK-N-BE(2) cells. **G** WB of the effect of *PIN1* depletion on SFPQ protein expression in CHP-134 and SK-N-BE(2) cells. **H** IF of the effect of *PIN1* depletion on SFPQ cellular localization in SK-N-BE(2) cells. Scale bar, 10 μm. **I** WB of the effect of *PIN1* depletion on SFPQ cellular localization. **J** Relative mRNA expression levels of *SOX2*, *TUJ1*, and *NDRG1*. *** *P* < 0.001, by two-tailed *t*-test. **K** IP and WB of the effect of TRIM59 on the interaction between SFPQ and PIN1. HA-tagged SFPQ, HA-tagged TRIM59, and Flag-tagged PIN1 were co-transfected into HEK293 cells. **L** IP of the interaction between SFPQ and PIN1 in SK-N-BE(2) cells transfected with shC or sh*TRIM59*

Given that TRIM59 functions as an E3 ubiquitin ligase [45], we initially explored its regulatory effects on SFPQ expression in NB cells. However, qRT PCR and western blot analyses showed that *TRIM59* depletion did not markedly alter SFPQ mRNA or protein levels (Fig. 5, A and B).

Previous studies indicate that beyond its established nuclear role, SFPQ also localizes to motor axons and facilitates motor neuron differentiation [46]. Based on this extranuclear distribution, we hypothesized that *TRIM59* depletion may affect SFPQ nuclear localization. To investigate this, we performed immunofluorescence (IF) analysis and found that *TRIM59* KD significantly reduced SFPQ nuclear localization and increased its cytosolic distribution in SK-N-BE(2) and CHP-134 cells (Fig. 5C). This redistribution was corroborated by nuclear-cytoplasmic fractionation, which showed decreased nuclear SFPQ levels accompanied by cytosolic accumulation upon *TRIM59* depletion (Fig. 5D). These findings provide evidence that TRIM59 contributes to the regulation of SFPQ nuclear localization.

Consistent with our previous study that the nuclear import of TRIM59 is mediated by the PIN1/importin α axis [32], we sought to determine whether this mechanism also applies to SFPQ. We first performed immunoprecipitation assays and found that both TRIM59 and SFPQ interact with the PIN1/importin α5 complex in NB cells (Fig. 5, E and F). Next, we knocked down the *PIN1* gene with two different shRNAs. Although *PIN1* depletion did not impair SFPQ protein expression (Fig. 5G), it significantly reduced the nuclear translocation of SFPQ, as confirmed by IF and WB analyses (Fig. 5, H and I). Similar to TRIM59 and SFPQ silencing, *PIN1* depletion extended neurite-like protrusions (Fig. 5H), upregulated *TUJ1* and *NDRG1* expression, and downregulated *SOX2* levels (Fig. 5J). Finally, we investigated the effect of TRIM59 on the interaction between SFPQ and PIN1. TRIM59 overexpression enhanced the association of SFPQ and PIN1 (Fig. 5K), whereas *TRIM59* KD decreased their association (Fig. 5L). Collectively, our data support the notion that TRIM59 modulates SFPQ nuclear translocation through the PIN1/importin α axis.

### TRIM59 facilitates PRMT1-catalyzed asymmetric di-methyl-arginine of SFPQ

**Fig. 6:**
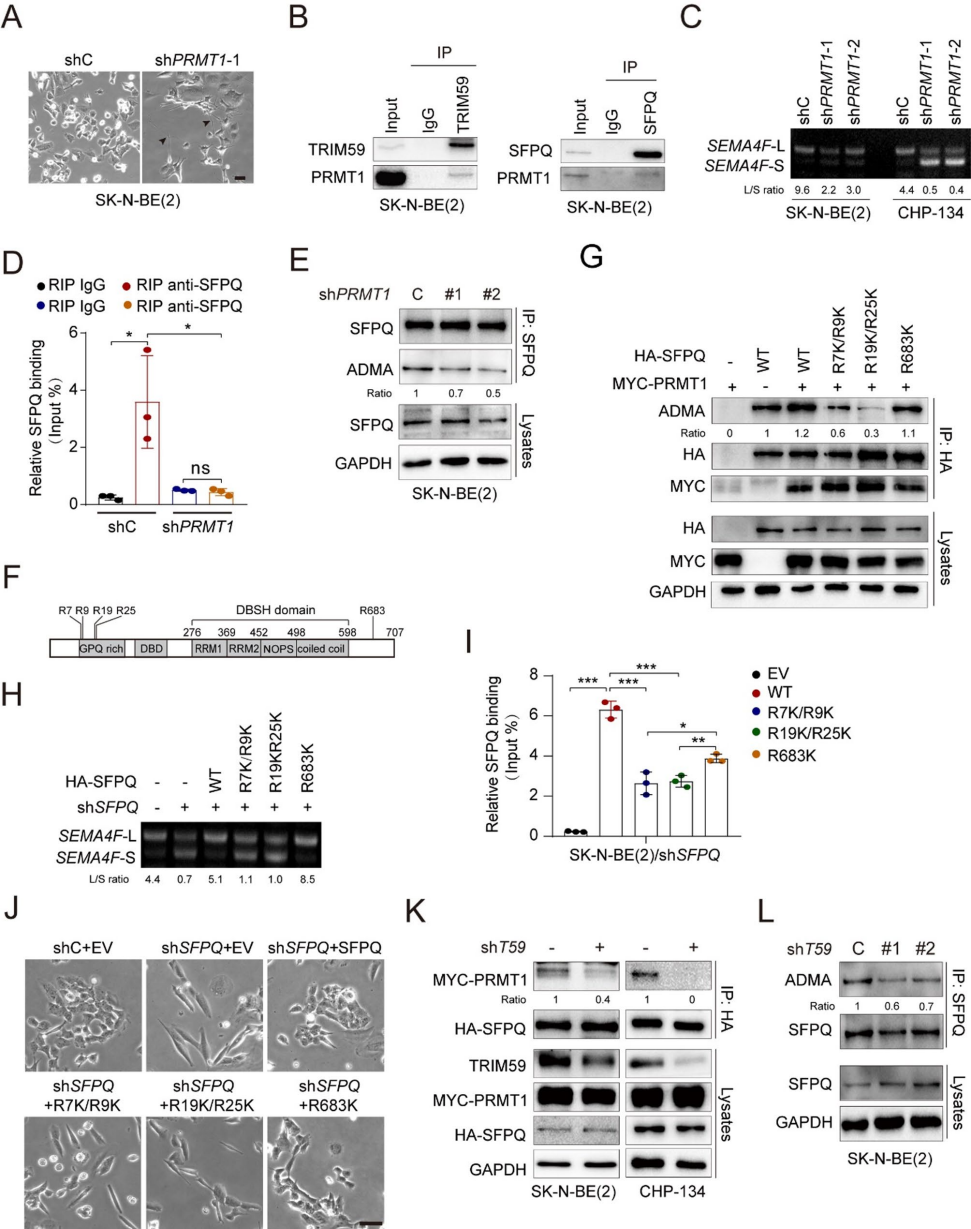
TRIM59 facilitates PRMT1-mediated asymmetric di-methyl-arginine of SFPQ. **A** Representative bright-field images of SK-N-BE(2) cells with shC or sh*PRMT1*−1. Scale bar, 50 μm. **B** IP of the interactions among TRIM59, SFPQ, and PRMT1 in SK-N-BE(2) cells. **C** RT-PCR of changes in *SEMA4F* splicing events in SK-N-BE(2) and CHP-134 cells with shC or sh*PRMT1.* **D **RIP of the association between SFPQ and *SEMA4F* pre-mRNAs following *PRMT1* depletion. **E **IP and WB of the effect of *PRMT1* KD on asymmetric di-methyl-arginine (ADMA) levels of SFPQ. SK-N-BE(2) cells with shC or sh*PRMT1* were immunoprecipitated with an anti-SFPQ antibody, followed by WB analysis using anti-ADMA and anti-SFPQ antibodies. **F** Schematic diagram of the arginine sites of SFPQ. **G **IP and WB to identify the asymmetric di-methyl-arginine sites of SFPQ. SK-N-BE(2) cells were transfected with MYC-tagged PRMT1 and HA-tagged wild-type SFPQ or methylation-deficient mutants (SFPQ-R7K/R9K, SFPQ-R19K/R25K, or SFPQ-R683K). Cell lysates were immunoprecipitated using an anti-HA antibody and analyzed by WB. **H** Effect of ectopic expression of wild-type (WT) SFPQ or its mutants on *SEMA4F* splicing. HA-tagged WT SFPQ or its mutants were re-expressed in SK-N-BE(2)/sh*SFPQ* cells. **I** RIP of the association between SFPQ and *SEMA4F* pre-mRNAs in SK-N-BE(2) cells with an empty vector (EV), HA-tagged wild-type, or methylation mutants as described in (f) using an anti-HA antibody. **J** Representative bright-field images of cells. **K** Effect of *TRIM59* KD on the interaction between SFPQ and PRMT1. **L** Effect of *TRIM59* KD on the asymmetric di-methyl-arginine levels of SFPQ. Data represent results from two or three independent experiments. Error bars indicate mean ± s.d. * *P* < 0.05, ** *P* < 0.01, *** *P* < 0.001, ns not significant, by two-tailed *t*-test

Protein arginine methyltransferases (PRMTs) are pivotal enzymes that catalyze the methylation of arginine residues, thereby influencing various biological processes, including RNA splicing and metabolic regulation [47]. Among the PRMT family, PRMT1 has been shown to catalyze the asymmetric dimethylation of arginine residues of SFPQ, promoting its association with mRNA [48]. Through mass spectrometry analysis, we identified PRMT1, PRMT3, and PRMT5 as potential TRIM59-interacting proteins (Supplemental Table 2). To investigate their functional relevance, we individually knocked down *PRMT1*, *PRMT3*, and *PRMT5* in SK-N-BE(2) cells (Supplemental Fig. 3A). Notably, *PRMT1* depletion mostly significantly enhanced neurite outgrowth and upregulated the expression of neuronal differentiation-related genes (Fig. 6A, and Supplemental Figure, 3B-D). The interaction of TRIM59 and SFPQ with PRMT1 in NBs was validated by reciprocal IP assays (Fig. 6B). Based on these findings, we hypothesized that TRIM59 regulates SFPQ methyl-arginine levels modulated by PRMT1, thereby influencing RNA splicing in NBs. To test this, we depleted *PRMT1* and observed a significant reduction in the ratio of *SEMA4F*-L to *SEMA4F*-S (Fig. 6C), along with diminished binding of SFPQ to *SEMA4F* pre-mRNAs (Fig. 6D).

Next, we investigated whether SFPQ could be methylated by PRMT1 in NBs. Control and *PRMT1* KD NB cells were immunoprecipitated with an anti-SFPQ antibody and analyzed by WB analysis using either anti-SFPQ or anti-ADMA (asymmetric di-methyl arginine motif (adme-R) multiMab) antibodies. As expected, *PRMT1* depletion significantly reduced SFPQ methylation levels (Fig. 6E). Mass spectrometry identified several arginine residues on SFPQ methylated by PRMT1, specifically R7, R9, R19, R25, and R683 [48]. To determine the significance of these residues, we constructed SFPQ mutants R7K/R9K, R19K/R25K, and R683K by substituting arginine (Arg) with lysine (Lys). The double mutations R7K/R9K and R19K/R25K nearly abolished PRMT1-catalyzed asymmetric di-methyl-arginine of SFPQ, while R683K mutant did not (Fig. 6, F and G). Additionally, the R7K/R9K and R19K/R25K double mutants were less effective in rescuing the AS changes in *SEMA4F* mRNA caused by *SFPQ* KD compared to wild-type (WT) SFPQ and the R683K mutant (Fig. 6H). RIP assays confirmed that the binding of the R7K/R9K and R19K/R25K double mutants to *SEMA4F* pre-mRNA was significantly weaker than that of WT SFPQ and the R683K mutant (Fig. 6I). We further evaluated the functional significance of these arginine methylation sites in NB. Unlike WT SFPQ and R683K mutant, the R7K/R9K and R19K/R25K double mutants failed to rescue the differentiation defects caused by *SFPQ* KD (Fig. 6J and Supplemental Fig. 3E). These data suggest that PRMT1 catalyzes the arginine methylation of SFPQ at R7/R9 and R19/R25 sites, which is essential for SFPQ-regulated AS of *SEMA4F* pre*-*mRNAs.

Finally, we assessed the role of TRIM59 in regulating SFPQ methyl-arginine levels modulated by PRMT1. *TRIM59* depletion inhibited the interaction between SFPQ and PRMT1 in both SK-N-BE(2) and CHP-134 cells by IP and WB assays (Fig. 6K). Moreover, *TRIM59* depletion decreased the asymmetric di-methyl-arginine levels of SFPQ (Fig. 6L). Collectively, these findings support the notion that TRIM59 regulates *SEMA4F* pre-mRNAs AS by modulating PRMT1-catalyzed asymmetric di-methyl-arginine levels of SFPQ.

### TRIM59-regulated NB differentiation is partially mediated by *SEMA4F* splicing switch

**Fig. 7:**
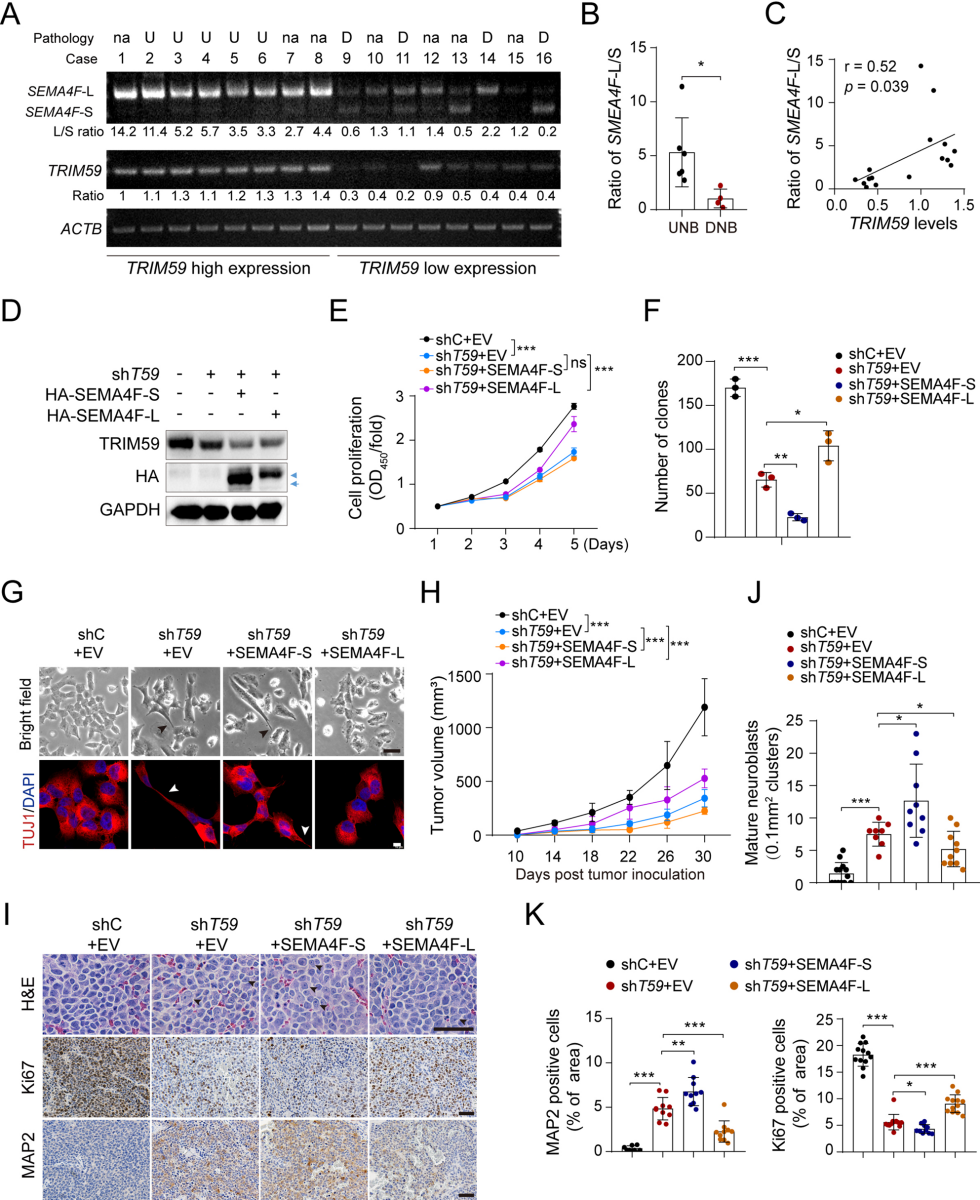
TRIM59-regulated NB differentiation is partially mediated by abnormal *SEMA4F* splicing switch. **A** RT-PCR of *SEMA4F* splicing events in NB tissues. U: Undifferentiated NBs; D: Differentiated NBs; na: not available. **B** The ratio of *SEMA4F*-L to *SEMA4F*-S isoform in undifferentiated cases (*n* = 6) and differentiated cases (*n* = 4). **C** Correlation between the *SEMA4F*-L/*SEMA4F*-S ratio and *TRIM59* expression in NB patient tumors. *p* = 0.039, by Spearman correlation test. **D** WB of the re-expression of SEMA4F-S and SEMA4F-L in SK-N-BE(2)/sh*TRIM59* cells. Arrow, target protein. **E** Effect of SEMA4F isoforms on cell proliferation in SK-N-BE(2)/sh*TRIM59* cells. **F **Quantification of survival colonies from Supplemental Figure 4E. **G** Representative bright-field images and TUJ1 IF analysis. Top panel: Scale bar, 50 µm. Bottom panel: Scale bar, 10 µm. **H **Tumor growth curves measured in xenograft mice. **I **H&E staining, along with Ki67 and MAP2 IHC staining, of xenograft tumor sections. Scale bar, 50 µm. **J **Quantification of mature neuroblasts in (I). **K** Quantification of Ki67- and MAP2-positive cells in (I). Data represent two or three independent experiments. Error bars indicate mean ± s.d.*P < 0.05, ** *P* < 0.01, *** *P* < 0.001, ns not significant, by two-tailed *t*-test

SEMA4F, a transmembrane semaphorin, regulates the outgrowth and migration of oligodendrocyte precursors and promotes their differentiation [49]. To explore the biological functions of *SEMA4F* isoforms, we assessed the mRNA expression levels of *SEMA4F*-S and *SEMA4F*-L in human NB tissues using RT-PCR. As shown in Fig. 7A and B, *SEMA4F*-L expression was notably reduced in clinical differentiated NBs compared to undifferentiated tumors from SCMC, while the opposite trend was observed for *SEMA4F*-S isoform. Moreover, a statistically significant positive correlation was observed between the *SEMA4F*-L/*SEMA4F*-S ratio and *TRIM59* expression (Fig. 7C).

To further elucidate the functions of *SEMA4F* isoforms, we exogenously overexpressed HA-tagged SEMA4F-S and SEMA4F-L in SK-N-BE(2) cells (Supplemental Fig. 4 A). Ectopic expression of SEMA4F-S, rather than SEMA4F-L, markedly inhibited cell proliferation (Supplemental Fig. 4B), promoted neurite outgrowth (Supplemental Figure, 4 C and D), and significantly upregulated the expression of neuronal differentiation-related genes (Supplemental Fig. 4E).

Finally, we investigated whether TRIM59-regulated NB differentiation is partially mediated by *SEMA4F* splicing switch. SEMA4F-S or SEMA4F-L was ectopically expressed in *TRIM59* KD SK-N-BE(2) cells (Fig. 7D). Exogenous expression of SEMA4F-L, but not SEMA4F-S, partially reversed *TRIM59* KD-reduced cell proliferation (Fig. 7E), colony formation (Fig. 7F and Supplemental Fig. 4 F), and *TRIM59* KD-increased NB differentiation (Fig. 7G and Supplemental Fig. 4G), highlighting the role of TRIM59-regulated *SEMA4F* alternative splicing in NB differentiation. We further explored the functional significance of *SEMA4F* isoforms in *TRIM59* KD-reduced tumorigenesis. Consistent with the in vitro findings, SMEA4F-L ectopic expression partially reversed the effects of *TRIM59* KD on subcutaneous tumor growth (Fig. 7H, and Supplemental Fig. 4, H and I) and NB differentiation phenotypes (Fig. 7, I-K). Overall, these findings indicate that TRIM59 regulates NB differentiation partially through the modulation of *SEMA4F* splicing switch.

### AAV-delivered *TRIM59*-targeting gRNA potentiates chemotherapeutic efficiency through enhanced NB tumor differentiation

**Fig. 8:**
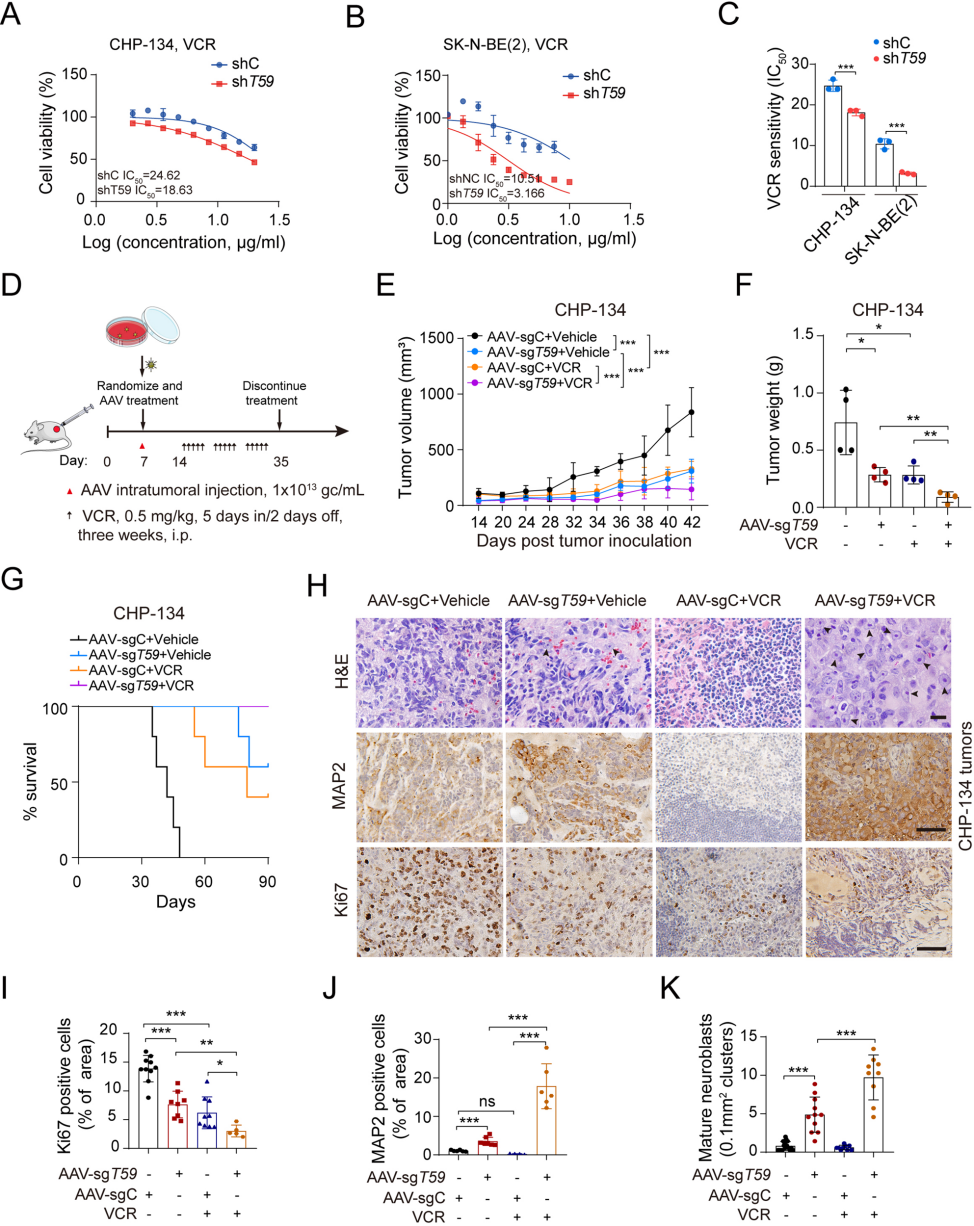
AAV-delivered *TRIM59*-targeting gRNA potentiates chemotherapeutic efficiency through enhanced NB tumor differentiation. **A **Dose-response curves showing the response of wild-type (WT) and *TRIM59* knockdown (KD) CHP-134 cells to vincristine (VCR). **B** Dose-response curves showing the response of WT and *TRIM59* KD SK-N-BE(2) cells to VCR. **C** Calculation of IC_50_ values for VCR in (A) and (B). **D** Schematic representation of therapeutic strategies combining AAV treatment with chemotherapy. Mice were randomly assigned to four experimental groups: (1) empty vector (EV), (2) AAV-*TRIM59* sgRNA (AAV-sg*T59*), (3) EV + VCR, and (4) AAV-sg*T59* + VCR. AAV treatment was administered on day 7 post-tumor implantation. VCR treatment (0.5 mg/kg in 1× PBS) was initiated 14 days post-tumor implantation and administered intraperitoneally 5 days per week for 3 weeks. **E** Tumor growth curves recorded in xenograft mice. **F **Quantification of tumor weight from Supplemental Figure 5E. **G **Kaplan-Meier survival curves of tumor xenograft studies. Subcutaneous tumors were allowed to grow to a maximum volume of 1500 mm^3^. *P* < 0.001, by the log-rank test. **H **H&E analysis, along with Ki67 and MAP2 IHC staining, of tumor sections from the tumors shown in Supplemental Figure 5E. Top panel: Scale bar, 20 µm. Middle and bottom panels: Scale bar, 50 µm. **I-K **Quantification of Ki67-positive cells **I**, MAP2-positive cells **J**, and mature neuroblasts **K**. Data represent three independent experiments. Error bars indicate mean ± s.d. *P < 0.05, ** *P* < 0.01, *** *P *< 0.001, ns not significant, by two-tailed *t*-test

Chemotherapy remains the primary therapeutic modality for NB patients [50]. However, its efficacy is often compromised by the development of multidrug resistance, particularly in high-risk cases [51]. We assessed whether *TRIM59* depletion synergizes with chemotherapeutic agents to reduce NB cell viability. Control and *TRIM59* KD NB cells were treated with vincristine (VCR), olaparib [a poly (ADP-ribose) polymerase (PARP) inhibitor], etoposide (VP-16), cyclophosphamide (CTX), or 13-cis retinoic acid (13-cRA). *TRIM59* KD significantly enhanced the sensitivity of SK-N-BE(2) and CHP-134 cells to VCR and olaparib (Fig. 8, A-C, and Supplemental Fig. 5B), while no substantial effect was observed with other chemotherapeutic agents in SK-N-BE(2) cells (Supplemental Fig. 5A).

To further elucidate the impact of *TRIM59* depletion on chemotherapy resistance, we developed an adeno-associated virus (AAV)-mediated Cas9 system to knockdown *TRIM59* (Supplemental Fig. 5, C and D), which showed no appreciable cytotoxicity in vitro (Supplemental Fig. 5, E and F). NB cells were implanted subcutaneously into NSG mice, which were randomly assigned on day 7 post-implantation to ensure equal tumor size across groups. Animals were then treated with control or targeting *TRIM59* AAV, VCR, or a combination (Fig. 8D).

As previously reported [44], VCR treatment significantly suppressed tumor proliferation (Fig. 8E, and Supplemental Fig. 5H), reduced tumor burden (Fig. 8F, and Supplemental Fig. 5, G, I, and J), and extended animal survival (Fig. 8G, and Supplemental Fig. 5K) compared to the control AAV (AAV-sgC) group. Consistently, VCR treatment markedly decreased Ki67 expression (Fig. 8, H and I, and Supplemental Fig. 5, L and M), without affecting MAP2 expression (Fig. 8, H and J, and Supplemental Fig. 5, L and N), or mature neuroblast formation (Fig. 8, H and K, and Supplemental Fig. 5, L and O). Treatment with *TRIM59* sgRNA AAV (AAV-sg*T59*) alone significantly reduced tumor proliferation and burden, enhanced tumor differentiation, and extended survival in tumor-bearing models (Fig. 8, E-K, and Supplemental Fig. 5, G-N). Notably, combination treatment with AAV-sg*T59* and VCR further suppressed tumor proliferation and burden (Fig. 8, E and F, and Supplemental Fig. 5, G-J). It is noteworthy that treatment with either AAV-sg*T59* virus or VCR alone results in only transient tumor growth control, whereas long-term remission was observed exclusively in the group receiving the combination therapy (Fig. 8G, and Supplemental Fig. 5K). Notably, tumor differentiation was significantly improved in the combined-treatment cohort compared to the AAV-sg*T59* virus-alone cohort (*p* < 0.001) (Fig. 8, H, J and K, and Supplemental Fig. 5, L-O). These data demonstrate that combining AAV-sg*T59* virus and VCR may improve chemotherapy efficacy and benefit patients with high-risk NBs.

### AAV-delivered TRIM59-gRNA boosts CAR-T therapy via *IRF1* splicing switch

**Fig. 9:**
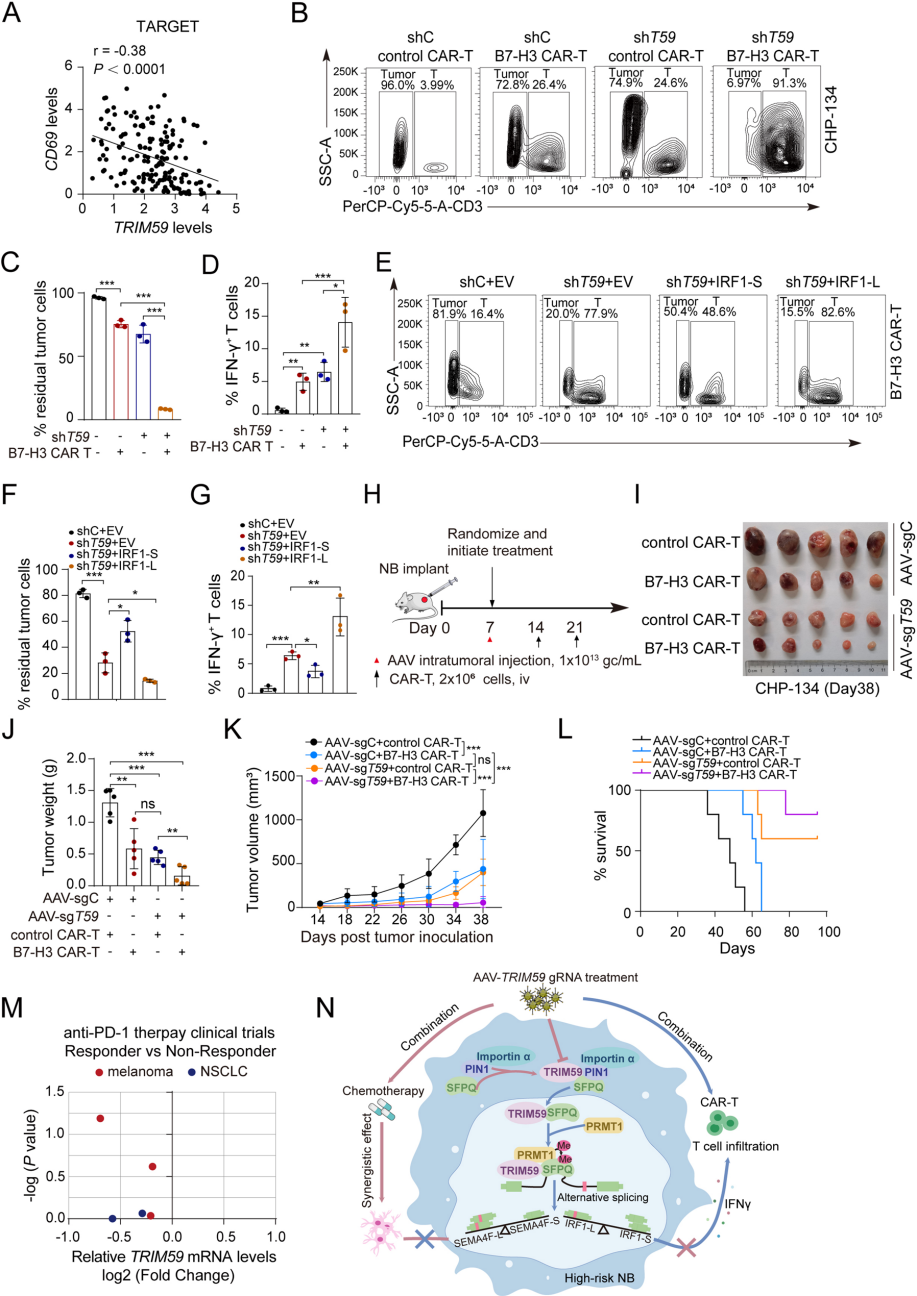
AAV-delivered *TRIM59*-gRNA boosts CAR-T therapy via *IRF1*-L isoform up-regulation. **A** Correlation between *CD69* and *TRIM59* expression in NB tumors from the TARGET dataset. *P* < 0.0001, by Spearman correlation test. **B** CHP-134 cells with shC or sh*TRIM59* were co-cultured with control CAR-T (CD19 CAR-T) or B7H3 CAR-T cells at an effector-to-target ratio of 1:3 for 72 h. Tumor cells (CD3^−^) and T cells (CD3^+^) were quantified by flow cytometry. **C** Quantification of the proportion of residual tumor cells in the co-culture experiments described in (B). **D **Quantification of the proportion of IFN-γ^+^ T cells in Supplemental Figure 6D. **E** CHP-134 cells with shC+EV, sh*TRIM59*+EV, sh*TRIM59*+IRF1-S, or sh*TRIM59*+IRF1-L, were co-cultured with B7H3 CAR-T cells at an effector-to-target ratio of 1:4 for 72 h. **F **Quantification of the proportion of residual tumor cells in (E). **G **Quantification of the proportion of IFN-γ^+^ T cells in (Supplemental Figure 6E). **H** Schematic representation of CAR T therapeutic strategies. **I** Images of tumors excised on day 38 post-transplantation from mice treated with AAV and CAR-T cells. **J **Quantification of tumor weight from (I). **K **Tumor growth curves recorded in xenograft mice. **L** Kaplan-Meier survival curves of tumor xenograft studies. The subcutaneous tumors were allowed to grow to a maximum volume of 1500 mm^3^.* P *< 0.001, by the log-rank test. **M** Analysis of RNA-seq datasets from three melanoma (GSE78220, PRJEB23709, and PHS000452) and two non-small cell lung cancer (NSCLC, GSE135222 and GSE126044). These anti-PD-1 trials revealed a positive correlation between low *TRIM59* expression and response, though not statistically significant. Red: Melanoma. Blue: NSCLC. **N** A working model of TRIM59 in alternative splicing and NB differentiation. Data represent two or three independent experiments. Error bars indicate mean ± s.d.* *P* < 0.05, ** *P* < 0.01, *** *P* < 0.001, by two-tailed *t*-test

IRF1 is a master transcriptional activator of interferon-γ (IFN-γ)-mediated signaling in cytotoxic T cell function [52], and disruption of IRF1 function impaired the activation of downstream immune-stimulating genes (IgSGs) and reduced T cell-mediated anti-tumor immunity in lung cancers [53]. Given that TRIM59 regulates *IRF1* pre-mRNA AS, we hypothesized that targeting TRIM59 could boost CAR-T therapy. Notably, analysis of the TARGET database revealed an inverse correlation between *TRIM59* expression and *CD69*, an early marker of T cell activation, in clinical NBs (Fig. 9A). Therefore, we investigated whether combining *TRIM59* KD with CAR-T cells could further increase the vulnerability of NBs to T cell killing. B7-H3 is highly expressed in NBs but minimally expressed in normal tissues, whereas CD19 is absent in NBs, making it an appropriate control for comparison [54, 55]. We generated chimeric antigen receptor (CAR) T cells targeting B7-H3 or CD19 (Supplemental Fig. 6A) and co-cultured *TRIM59* KD CHP-134 and SK-N-BE(2) cells with activated B7-H3 CAR-T cells. Strikingly, > 93% of CHP-134 cells and > 89% of SK-N-BE(2) cells were killed following *TRIM59* KD and treatment with B7-H3 CAR-T cells, whereas only < 25% of CHP-134 cells and < 55% of SK-N-BE(2) cells were killed in the presence of *TRIM59* KD and control CD19 CAR-T cells (Fig. 9, B and C, and Supplemental Fig. 6, B and C). Moreover, a significant increase in IFN-γ^+^ CAR-T cells was observed in the *TRIM59* KD groups, particularly in the B7-H3 CAR-T treatment group, compared to WT groups (Fig. 9D and Supplemental Fig. 6D). These results suggest that *TRIM59* KD may enhance the cytotoxic activity of CAR-T cells, thereby improving their therapeutic efficacy in vitro.

Next, we investigated whether TRIM59-regulated CAR-T cell activity is partially mediated by *IRF1* splicing switch. IRF1-S or IRF1-L was ectopically expressed in *TRIM59* KD CHP-134 cells (Supplemental Fig. 6E). Exogenous expression of IRF1-S, but not IRF1-L, partially reversed *TRIM59* KD-enhanced B7-H3 CAR-T cell killing efficacy (Fig. 9, E and F) and increased IFN-γ^+^ CAR-T cell ratio (Fig. 9G, and Supplemental Fig. 6E). Notably, ectopic expression of IRF1-L isoform further augmented CAR-T cell cytotoxic activity and IFN-γ^+^ CAR-T cell percentage (Fig. 9, E-G, and Supplemental Fig. 6 F). These data support that *TRIM59* KD activates CAR-T cell activity via *IRF1* splicing switch.

We further injected CHP-134 cells into NSG mice and initiated the treatment with AAV-sg*T59* or control AAV-sgC virus on day 7, followed by treatment with B7-H3 or control CAR-T cells on day 14 and day 21 (Fig. 9H). Compared to treatment with AAV-sg*T59* virus and control CAR-T cell alone, the combination treatment of AAV-sg*T59* virus and B7-H3 CAR-T cells markedly reduced tumor cell proliferation and tumor burden (Fig. 9, I-K). B7-H3 CAR-T cell treatment reduced Ki67 expression (Supplemental Figure, 6G and H) but did not significantly enhance the NB differentiation induced by AAV-sg*T59* virus treatment (Supplemental Fig. 6, F and G). In addition, long-term remission was more frequently in the combination treatment group compared to either AAV or CAR-T cells monotherapy (Fig. 9L).

Finally, we interrogated RNA-seq datasets from anti-PD-1 antibody-treated patients across three melanoma and two non-small-cell lung cancer (NSCLC) clinical trials. Notably, low *TRIM59* expression was positively correlated with anti-PD-1 response, albeit not significant (Fig. 9M). Taken together, these data provide preclinical evidence that targeting TRIM59 may boost immunotherapeutic efficacy in high-risk NBs.

## Discussion

In this study, we establish TRIM59 as a critical regulator of NB differentiation through its modulation of *SEMA4F* pre-mRNA splicing (Fig. 9N). Integrative analysis of multi-cohort NB transcriptomic datasets identifies TRIM59 as an independent prognostic marker, with high expression correlating with undifferentiated histopathology, chemoresistance, and impaired tumor-infiltrating T cell activity. Mechanistically, *TRIM59* depletion drives NB xenograft differentiation toward a mature neuroblast-like phenotype, concomitant with activation of differentiation-related signaling pathways. Furthermore, *TRIM59*-targeting AAV-gRNA synergizes with vincristine by augmenting NB differentiation, while *TRIM59* knockdown enhances anti-tumor immunity via *IRF1* splicing-dependent regulation of T cell function. Clinical validation across multiple cancer cohorts reveals that elevated *TRIM59* expression predicts resistance to anti-PD-1 immunotherapy, although the difference did not reach statistical significance, underscoring its role in immune evasion. Collectively, our findings position TRIM59 as a novel therapeutic target for high-risk neuroblastoma, with dual utility in overcoming differentiation blockade and restoring immune-mediated tumor control.

We uncovered a novel function of TRIM59 as a critical regulator of RNA splicing. While TRIM59 is primarily recognized as an E3 ubiquitin ligase involved in immunity, immune-related diseases, and various cancers [40, 42, 56, 57]. This study demonstrates that TRIM59 regulates pre-mRNA AS of genes involved in tumorigenesis and differentiation through its interaction with SFPQ.

To the best of our knowledge, this study is the first to identify the regulatory factors controlling SFPQ nucleocytoplasmic transport. SFPQ is a critical regulator of RNA splicing, essential for neuronal function and implicated neurodegenerative processes [58–60]. Its precise nucleocytoplasmic localization is critical for proper neuronal development and cellular homeostasis [61]. Previous studies have shown that elevated zinc levels induce SFPQ cytoplasmic aggregates in primary neurons, promoting neuronal differentiation and altering its RNA-binding properties [46, 62]. Interestingly, SFPQ has also been detected in motor axons, where it plays a key role in axon maturation and connectivity [63]. However, the molecular mechanism underlying SFPQ nuclear translocation has remained unclear. Here, we reveal that TRIM59 regulates this process via the PIN1/Importin α axis. *TRIM59* depletion impairs SFPQ nuclear translocation, leading to cytoplasmic aggregates and promoting neuronal differentiation in NBs.

RNA splicing factors are extensively modified by PRMTs, and inhibiting PRMTs has emerged as a promising strategy to overcome cancer resistance [64, 65]. PRMT1, the primary type I PRMT, regulates the arginine methylation of SFPQ, enhancing its association with mRNA [48]. Here, we demonstrate that TRIM59 augments PRMT1-mediated arginine methylation of SFPQ in NBs. Beyond the established roles of PRMT1 in SFPQ methylation and RNA binding, our study identifies specific methylation sites on SFPQ that are regulated by PRMT1, providing new insights into the mechanism of RNA splicing regulation in NBs.

SEMA4F is a member of the transmembrane protein family, involved in neural development and adult brain plasticity [49]. In the peripheral nervous system (PNS), SEMA4F maintains Schwann cell-axon interaction by downregulating the Ras/Raf/ERK pathway [66]. A previous study has shown that SEMA4F drives the progression from low-grade gliomas to high-grade GBM by mediating tumor cell proliferation and infiltration, which are dependent on remote neuronal activity [67]. Our findings demonstrate that *TRIM59* depletion upregulates the short isoform of *SEMA4F* (*SEMA4F-*S) and downregulates the long isoform (*SEMA4F-*L). Both *TRIM59* depletion and the *SEMA4F*-S variant share similar functions in enhancing NB differentiation. Furthermore, the tumor-suppressive effects of *TRIM59* depletion could be partially rescued by overexpression of SEMA4F*-*L. Clinically, TRIM59-mediated *SEMA4F* splicing is supported by the observed increase in the *SEMA4F*-L/*SEMA4F*-S ratio in poorly differentiated NB compared to well-differentiated specimens.

High-risk NB is known for its insensitivity to differentiation therapy and is prone to therapeutic resistance [35]. The current treatment regimen for high-risk NB patients consists of three stages: induction, consolidation, and maintenance [68]. While RA combined with chemotherapy benefits some high-risk NB patients, its efficacy remains limited [36, 37]. In this study, targeting TRIM59 not only markedly promotes NB cell differentiation toward mature neuroblasts in xenograft models, but also synergizes with chemotherapy to enhance differentiation and reduce tumor burden, suggesting TRIM59 as a potential target for clinical differentiation therapy.

CAR-T cell therapy exhibits limited efficacy in high-risk NBs [69, 70]. Here, we demonstrate that targeting TRIM59 significantly enhances NB sensitivity to B7-H3 CAR-T therapy via mediating *IRF1* pre-mRNA splicing. Notably, data from various cancer clinical trials indicate that lower expression of *TRIM59* is positively correlated with a better response to immune checkpoint blockade therapy, albeit not significantly. A previous study indicated that SFPQ regulates *IRF1* pre-mRNA alternative splicing in mouse T cells [43]. Consistent with this, we further demonstrate that TRIM59-regulated SFPQ modulates human *IRF1* alternative splicing.

Although our results demonstrate that *TRIM59*-targeting AAV effectively enhances NB sensitivity to VCR and improves CAR-T cell therapy outcomes, the efficacy of this approach in other therapeutic contexts remains uncertain. While TRIM59 inhibition holds promise in promoting differentiation and boosting anti-tumor immunity in preclinical models, its long-term effects and potential side effects have not been thoroughly evaluated. The safety and tolerability of TRIM59 inhibition, particularly across different clinical subpopulations, require further investigation. Future research must focus on refining these strategies and assessing their efficacy, generalizability and long-term clinical applicability across various malignancies.

## Conclusion

In conclusion, this study uncovers a previously unrecognized role for TRIM59 in RNA splicing, NB differentiation, and therapeutic resistance. Our study demonstrates that *TRIM59*-targeting AAV synergizes with chemotherapy or CAR-T cells to more effectively suppress NB progression compared to monotherapies. This clinically feasible combination strategy represents a promising translatable approach for improving outcomes in high-risk NB patients.

## Supplementary Information

Supplementary Material 1.

Supplementary Material 2.

Supplementary Material 3.

## Data Availability

RNA-seq data reported in this study have been deposited in the Genome Sequence Archive in National Genomics Data Center, China National Center for Bioinformation/Beijing Institute of Genomics, Chinese Academy of Sciences (GSA-Human: HRA002060 and HRA009481) that are publicly accessible at [***https://ngdc.cncb.ac.cn/gsa-human***](https:/ngdc.cncb.ac.cn/gsa-human). The data supporting the findings of this study are available within the article and its Supporting Information files or available from the corresponding author upon reasonable request.

## Abbreviations

NB: Neuroblastoma
TRIM: The tripartite-motif family
RA: Retinoic acids
ATRA: All-trans-retinoic acid
13-cRA: 13-cis-retinoic acid
AS: Alternative splicing
AAV: Adeno-associated virus
VCR: Vincristine
GNBs: Ganglioneuroblastomas
PLA: Proximity ligation assay
rMATS: Replicate Multivariate Analysis of Transcript Splicing
PRMTs: Protein arginine methyltransferases
ADMA: Asymmetric di-methyl arginine motif (adme-R) multiMab
GO: Gene Ontology

## References

[1] Yu EY, Cheung NV, Lue NF. Connecting telomere maintenance and regulation to the developmental origin and differentiation States of neuroblastoma tumor cells. J Hematol Oncol. 2022;15(1):117. 36030273 10.1186/s13045-022-01337-w PMC9420296

[2] Boeva V, Louis-Brennetot C, Peltier A, Durand S, Pierre-Eugène C, Raynal V, Etchevers HC, Thomas S, Lermine A, Daudigeos-Dubus E, et al. Heterogeneity of neuroblastoma cell identity defined by transcriptional circuitries. Nat Genet. 2017;49(9):1408–13. 28740262 10.1038/ng.3921

[3] Brodeur GM. Neuroblastoma: biological insights into a clinical enigma. Nat Rev Cancer. 2003;3(3):203–16. 12612655 10.1038/nrc1014

[4] Ben Amar D, Thoinet K, Villalard B, Imbaud O, Costechareyre C, Jarrosson L, Reynaud F, Ducassou N, Couté J, Brunet Y. Environmental cues from neural crest derivatives act as metastatic triggers in an embryonic neuroblastoma model. Nat Commun. 2022;13(1):2549. 35538114 10.1038/s41467-022-30237-3 PMC9091272

[5] Dong R, Yang R, Zhan Y, Lai HD, Ye CJ, Yao XY, Luo WQ, Cheng XM, Miao JJ, Wang JF, et al. Single-Cell characterization of malignant phenotypes and developmental trajectories of adrenal neuroblastoma. Cancer Cell. 2020;38(5):716–33. 32946775 10.1016/j.ccell.2020.08.014

[6] Reynolds CP, Matthay KK, Villablanca JG, Maurer BJ. Retinoid therapy of high-risk neuroblastoma. Cancer Lett. 2003;197(1–2):185–92. 12880980 10.1016/s0304-3835(03)00108-3

[7] Bagatell R, Park JR, Acharya S, Aldrink J, Allison J, Alva E, Arndt C, Benedetti D, Brown E, Cho S, Neuroblastoma, et al. Version 2.2024, NCCN clinical practice guidelines in oncology. J Natl Compr Canc Netw. 2024;22(6):413–33. 39151455 10.6004/jnccn.2024.0040

[8] Lampis S, Raieli S, Montemurro L, Bartolucci D, Amadesi C, Bortolotti S, Angelucci S, Scardovi AL, Nieddu G, Cerisoli L, et al. The MYCN inhibitor BGA002 restores the retinoic acid response leading to differentiation or apoptosis by the mTOR block in MYCN-amplified neuroblastoma. J Exp Clin Cancer Res. 2022;41(1):160. 35490242 10.1186/s13046-022-02367-5 PMC9055702

[9] Maeshima R, Moulding D, Stoker AW, Hart SL. MYCN Silencing by RNAi induces neurogenesis and suppresses proliferation in models of neuroblastoma with resistance to retinoic acid. Nucleic Acid Ther. 2020;30(4):237–48. 32240058 10.1089/nat.2019.0831 PMC7415885

[10] Bradley RK, Anczuków O. RNA splicing dysregulation and the hallmarks of cancer. Nat Rev Cancer. 2023;23(3):135–55. 36627445 10.1038/s41568-022-00541-7 PMC10132032

[11] Nikom D, Zheng S. Alternative splicing in neurodegenerative disease and the promise of RNA therapies. Nat Rev Neurosci. 2023;24(8):457–73. 37336982 10.1038/s41583-023-00717-6

[12] Rogalska ME, Vivori C, Valcárcel J. Regulation of pre-mRNA splicing: roles in physiology and disease, and therapeutic prospects. Nat Rev Genet. 2023;24(4):251–69. 36526860 10.1038/s41576-022-00556-8

[13] Bonnal SC, López-Oreja I, Valcárcel J. Roles and mechanisms of alternative splicing in cancer - implications for care. Nat Rev Clin Oncol. 2020;17(8):457–74. 32303702 10.1038/s41571-020-0350-x

[14] Nijhuis A, Sikka A, Yogev O, Herendi L, Balcells C, Ma Y, Poon E, Eckold C, Valbuena GN, Xu Y, et al. Indisulam targets RNA splicing and metabolism to serve as a therapeutic strategy for high-risk neuroblastoma. Nat Commun. 2022;13(1):1380. 35296644 10.1038/s41467-022-28907-3 PMC8927615

[15] Singh S, Quarni W, Goralski M, Wan S, Jin H, Van de Velde LA, Fang J, Wu Q, Abu-Zaid A, Wang T, et al. Targeting the spliceosome through RBM39 degradation results in exceptional responses in high-risk neuroblastoma models. Sci Adv. 2021;7(47):eabj5405. 34788094 10.1126/sciadv.abj5405 PMC8598007

[16] Choi SH, Flamand MN, Liu B, Zhu H, Hu M, Wang M, Sewell J, Holley CL, Al-Hashimi HM, Meyer KD. RBM45 is an m(6)A-binding protein that affects neuronal differentiation and the splicing of a subset of mRNAs. Cell Rep. 2022;40(9):111293. 36044854 10.1016/j.celrep.2022.111293 PMC9472474

[17] Chen J, Hackett CS, Zhang S, Song YK, Bell RJ, Molinaro AM, Quigley DA, Balmain A, Song JS, Costello JF, et al. The genetics of splicing in neuroblastoma. Cancer Discov. 2015;5(4):380–95. 25637275 10.1158/2159-8290.CD-14-0892 PMC4390477

[18] Zhang S, Wei JS, Li SQ, Badgett TC, Song YK, Agarwal S, Coarfa C, Tolman C, Hurd L, Liao H, et al. MYCN controls an alternative RNA splicing program in high-risk metastatic neuroblastoma. Cancer Lett. 2016;371(2):214–24. 26683771 10.1016/j.canlet.2015.11.045 PMC4738031

[19] Shi Y, Yuan J, Rraklli V, Maxymovitz E, Cipullo M, Liu M, Li S, Westerlund I, Bedoya-Reina OC, Bullova P, et al. Aberrant splicing in neuroblastoma generates RNA-fusion transcripts and provides vulnerability to spliceosome inhibitors. Nucleic Acids Res. 2021;49(5):2509–21. 33555349 10.1093/nar/gkab054 PMC7969022

[20] Ozato K, Shin DM, Chang TH, Morse HC. 3rd. TRIM family proteins and their emerging roles in innate immunity. Nat Rev Immunol. 2008;8(11):849–60. 18836477 10.1038/nri2413 PMC3433745

[21] Cai C, Tang YD, Zhai J, Zheng C. The RING finger protein family in health and disease. Signal Transduct Target Ther. 2022;7(1):300. 36042206 10.1038/s41392-022-01152-2 PMC9424811

[22] Hatakeyama STRIM, Family Proteins. Roles in Autophagy, Immunity, and carcinogenesis. Trends Biochem Sci. 2017;42(4):297–311. 28118948 10.1016/j.tibs.2017.01.002

[23] Schwamborn JC, Berezikov E, Knoblich JA. The TRIM-NHL protein TRIM32 activates MicroRNAs and prevents self-renewal in mouse neural progenitors. Cell. 2009;136(5):913–25. 19269368 10.1016/j.cell.2008.12.024 PMC2988196

[24] Liu RX, Gu RH, Li ZP, Hao ZQ, Hu QX, Li ZY, Wang XG, Tang W, Wang XH, Zeng YK, et al. Trim21 depletion alleviates bone loss in osteoporosis via activation of YAP1/β-catenin signaling. Bone Res. 2023;11(1):56. 37884520 10.1038/s41413-023-00296-3 PMC10603047

[25] Nguyen DTT, Richter D, Michel G, Mitschka S, Kolanus W, Cuevas E, Wulczyn FG. The ubiquitin ligase LIN41/TRIM71 targets p53 to antagonize cell death and differentiation pathways during stem cell differentiation. Cell Death Differ. 2017;24(6):1063–78. 28430184 10.1038/cdd.2017.54 PMC5442473

[26] Chen Y, Xu X, Ding K, Tang T, Cai F, Zhang H, Chen Z, Qi Y, Fu Z, Zhu G, et al. TRIM25 promotes glioblastoma cell growth and invasion via regulation of the PRMT1/c-MYC pathway by targeting the splicing factor NONO. J Exp Clin Cancer Res. 2024;43(1):39. 38303029 10.1186/s13046-024-02964-6 PMC10835844

[27] Su X, Wu C, Ye X, Zeng M, Zhang Z, Che Y, Zhang Y, Liu L, Lin Y, Yang R. Embryonic lethality in mice lacking Trim59 due to impaired gastrulation development. Cell Death Dis. 2018;9(3):302. 29467473 10.1038/s41419-018-0370-y PMC5833458

[28] Fan L, Gong Y, He Y, Gao WQ, Dong X, Dong B, Zhu HH, Xue W. TRIM59 is suppressed by androgen receptor and acts to promote lineage plasticity and treatment-induced neuroendocrine differentiation in prostate cancer. Oncogene. 2023;42(8):559–71. 36544044 10.1038/s41388-022-02498-1

[29] Lou M, Gao Z, Zhu T, Mao X, Wang Y, Yuan K, Tong J. TRIM59 as a novel molecular biomarker to predict the prognosis of patients with NSCLC. Oncol Lett. 2020;19(2):1400–8. 31966070 10.3892/ol.2019.11199 PMC6956412

[30] Chen G, Chen W, Ye M, Tan W, Jia B. TRIM59 knockdown inhibits cell proliferation by down-regulating the Wnt/β-catenin signaling pathway in neuroblastoma. Biosci Rep. 2019;39(1):BSR20181277. 30389710 10.1042/BSR20181277 PMC6340953

[31] Liu Y, Jiang N, Chen W, Zhang W, Shen X, Jia B, Chen G. TRIM59-mediated ferroptosis enhances neuroblastoma development and chemosensitivity through p53 ubiquitination and degradation. Heliyon. 2024;10(4):e26014. 38434050 10.1016/j.heliyon.2024.e26014 PMC10906161

[32] Sang Y, Li Y, Song L, Alvarez AA, Zhang W, Lv D, Tang J, Liu F, Chang Z, Hatakeyama S, et al. TRIM59 promotes gliomagenesis by inhibiting TC45 dephosphorylation of STAT3. Cancer Res. 2018;78(7):1792–804. 29386185 10.1158/0008-5472.CAN-17-2774 PMC5882560

[33] Wang T, Wan X, Yang F, Shi W, Liu R, Ding L, Tang Y, Luo C, Yang X, Ma Y, et al. Successful treatment of TCF3-HLF-positive childhood B-ALL with chimeric antigen receptor T-Cell therapy. Clin Lymphoma Myeloma Leuk. 2021;21(6):386–92. 33640284 10.1016/j.clml.2021.01.014

[34] Hu Z, Wang S, Zhang C, Gao N, Li M, Wang D, Wang D, Liu D, Liu H, Ong SG, et al. A compact Cas9 ortholog from Staphylococcus auricularis (SauriCas9) expands the DNA targeting scope. PLoS Biol. 2020;18(3):e3000686. 32226015 10.1371/journal.pbio.3000686 PMC7145270

[35] Matthay KK, Reynolds CP, Seeger RC, Shimada H, Adkins ES, Haas-Kogan D, Gerbing RB, London WB, Villablanca JG. Long-term results for children with high-risk neuroblastoma treated on a randomized trial of myeloablative therapy followed by 13-cis-retinoic acid: a children’s oncology group study. J Clin Oncol. 2009;27(7):1007–13. 19171716 10.1200/JCO.2007.13.8925 PMC2738615

[36] Villablanca JG, Khan AA, Avramis VI, Seeger RC, Matthay KK, Ramsay NK, Reynolds CP. Phase I trial of 13-cis-retinoic acid in children with neuroblastoma following bone marrow transplantation. J Clin Oncol. 1995;13(4):894–901. 7707116 10.1200/JCO.1995.13.4.894

[37] Matthay KK, Villablanca JG, Seeger RC, Stram DO, Harris RE, Ramsay NK, Swift P, Shimada H, Black CT, Brodeur GM, et al. Treatment of high-risk neuroblastoma with intensive chemotherapy, radiotherapy, autologous bone marrow transplantation, and 13-cis-retinoic acid. Children’s cancer group. N Engl J Med. 1999;341(16):1165–73. 10519894 10.1056/NEJM199910143411601

[38] Cheung NK, Cheung IY, Kushner BH, Ostrovnaya I, Chamberlain E, Kramer K, Modak S. Murine anti-GD2 monoclonal antibody 3F8 combined with granulocyte-macrophage colony-stimulating factor and 13-cis-retinoic acid in high-risk patients with stage 4 neuroblastoma in first remission. J Clin Oncol. 2012;30(26):3264–70. 22869886 10.1200/JCO.2011.41.3807 PMC3434986

[39] Zhou MJ, Doral MY, DuBois SG, Villablanca JG, Yanik GA, Matthay KK. Different outcomes for relapsed versus refractory neuroblastoma after therapy with (131)I-metaiodobenzylguanidine ((131)I-MIBG). Eur J Cancer. 2015;51(16):2465–72. 26254811 10.1016/j.ejca.2015.07.023 PMC4607645

[40] Sang Y, Li Y, Zhang Y, Alvarez AA, Yu B, Zhang W, Hu B, Cheng SY, Feng H. CDK5-dependent phosphorylation and nuclear translocation of TRIM59 promotes macroH2A1 ubiquitination and tumorigenicity. Nat Commun. 2019;10(1):4013. 31488827 10.1038/s41467-019-12001-2 PMC6728346

[41] Liang M, Chen X, Wang L, Qin L, Wang H, Sun Z, Zhao W, Geng B. Cancer-derived Exosomal TRIM59 regulates macrophage NLRP3 inflammasome activation to promote lung cancer progression. J Exp Clin Cancer Res. 2020;39(1):176. 32867817 10.1186/s13046-020-01688-7 PMC7457778

[42] Zhou Z, Ji Z, Wang Y, Li J, Cao H, Zhu HH, Gao WQ. TRIM59 is up-regulated in gastric tumors, promoting ubiquitination and degradation of p53. Gastroenterology. 2014;147(5):1043–54. 25046164 10.1053/j.gastro.2014.07.021

[43] Bernard A, Hibos C, Richard C, Viltard E, Chevrier S, Lemoine S, Melin J, Humblin E, Mary R, Accogli T, et al. The tumor microenvironment impairs Th1 IFNγ secretion through alternative splicing modifications of Irf1 Pre-mRNA. Cancer Immunol Res. 2021;9(3):324–36. 33419764 10.1158/2326-6066.CIR-19-0679

[44] Yasuhara T, Xing YH, Bauer NC, Lee L, Dong R, Yadav T, Soberman RJ, Rivera MN, Zou L. Condensates induced by transcription Inhibition localize active chromatin to nucleoli. Mol Cell. 2022;82(15):2738–53. 35662392 10.1016/j.molcel.2022.05.010 PMC9357099

[45] Tan P, He L, Zhou Y. TRIM59 deficiency curtails breast cancer metastasis through SQSTM1-selective autophagic degradation of PDCD10. Autophagy. 2019;15(4):747–9. 30653426 10.1080/15548627.2019.1569951 PMC6526857

[46] Widagdo J, Udagedara S, Bhembre N, Tan JZA, Neureiter L, Huang J, Anggono V, Lee M. Familial ALS-associated SFPQ variants promote the formation of SFPQ cytoplasmic aggregates in primary neurons. Open Biol. 2022;12(9):220187. 36168806 10.1098/rsob.220187 PMC9516340

[47] Guccione E, Richard S. The regulation, functions and clinical relevance of arginine methylation. Nat Rev Mol Cell Biol. 2019;20(10):642–57. 31350521 10.1038/s41580-019-0155-x

[48] Snijders AP, Hautbergue GM, Bloom A, Williamson JC, Minshull TC, Phillips HL, Mihaylov SR, Gjerde DT, Hornby DP, Wilson SA, et al. Arginine methylation and citrullination of splicing factor proline- and glutamine-rich (SFPQ/PSF) regulates its association with mRNA. RNA. 2015;21(3):347–59. 25605962 10.1261/rna.045138.114 PMC4338332

[49] Armendáriz BG, Bribian A, Pérez-Martínez E, Martínez A, de Castro F, Soriano E, Burgaya F. Expression of semaphorin 4F in neurons and brain oligodendrocytes and the regulation of oligodendrocyte precursor migration in the optic nerve. Mol Cell Neurosci. 2012;49(1):54–67. 21945643 10.1016/j.mcn.2011.09.003

[50] Pinto NR, Applebaum MA, Volchenboum SL, Matthay KK, London WB, Ambros PF, Nakagawara A, Berthold F, Schleiermacher G, Park JR, et al. Advances in risk classification and treatment strategies for neuroblastoma. J Clin Oncol. 2015;33(27):3008–17. 26304901 10.1200/JCO.2014.59.4648 PMC4567703

[51] Grossmann LD, Chen CH, Uzun Y, Thadi A, Wolpaw AJ, Louault K, Goldstein Y, Surrey LF, Martinez D, Calafatti M, et al. Identification and characterization of chemotherapy resistant high-risk neuroblastoma persister cells. Cancer Discov. 2024;14(12):2387–406. 39083807 10.1158/2159-8290.CD-24-0046 PMC11609622

[52] Liu J, Guan X, Ma X. Interferon regulatory factor 1 is an essential and direct transcriptional activator for interferon {gamma}-induced RANTES/CCl5 expression in macrophages. J Biol Chem. 2005;280(26):24347–55. 15860458 10.1074/jbc.M500973200

[53] Alburquerque-Bejar JJ, Navajas-Chocarro P, Saigi M, Ferrero-Andres A, Morillas JM, Vilarrubi A, Gomez A, Mate JL, Munoz-Marmol AM, Romero OA, et al. MYC activation impairs cell-intrinsic IFNγ signaling and confers resistance to anti-PD1/PD-L1 therapy in lung cancer. Cell Rep Med. 2023;4(4):101006. 37044092 10.1016/j.xcrm.2023.101006 PMC10140599

[54] Zeng L, Xu H, Li SH, Xu SY, Chen K, Qin LJ, Miao L, Wang F, Deng L, Wang FH, et al. Cross-cohort analysis identified an immune checkpoint-based signature to predict the clinical outcomes of neuroblastoma. J Immunother Cancer. 2023;11(5):11. 10.1136/jitc-2022-005980 PMC10163522 37130627

[55] Bergaggio E, Tai WT, Aroldi A, Mecca C, Landoni E, Nüesch M, Mota I, Metovic J, Molinaro L, Ma L, et al. ALK inhibitors increase ALK expression and sensitize neuroblastoma cells to ALK.CAR-T cells. Cancer Cell. 2023;41(12):2100–16. 38039964 10.1016/j.ccell.2023.11.004 PMC10793157

[56] Li X, Pan M, Tian X, Yang LZ, Zhang J, Yan D, Xu B, Zhao L, Fang W. Myeloid cell Trim59 deficiency worsens experimental ischemic stroke and alters cerebral proteomic profile. J Inflamm Res. 2024;17:4827–43. 39051047 10.2147/JIR.S469651 PMC11268786

[57] Wang H, Lou J, Liu H, Liu Y, Xie B, Zhang W, Xie J, Pan H, Han W. TRIM59 deficiency promotes M1 macrophage activation and inhibits colorectal cancer through the STAT1 signaling pathway. Sci Rep. 2024;14(1):16081. 38992114 10.1038/s41598-024-66388-0 PMC11239810

[58] Belur NR, Bustos BI, Lubbe SJ, Mazzulli JR. Nuclear aggregates of NONO/SFPQ and A-to-I-edited RNA in parkinson’s disease and dementia with lewy bodies. Neuron. 2024;112(15):2558–80. 38761794 10.1016/j.neuron.2024.05.003 PMC11309915

[59] Taylor R, Hamid F, Fielding T, Gordon PM, Maloney M, Makeyev EV, Houart C. Prematurely terminated intron-retaining mRNAs invade axons in SFPQ null-driven neurodegeneration and are a hallmark of ALS. Nat Commun. 2022;13(1):6994. 36414621 10.1038/s41467-022-34331-4 PMC9681851

[60] Fukuda Y, Pazyra-Murphy MF, Silagi ES, Tasdemir-Yilmaz OE, Li Y, Rose L, Yeoh ZC, Vangos NE, Geffken EA, Seo HS, et al. Binding and transport of SFPQ-RNA granules by KIF5A/KLC1 motors promotes axon survival. J Cell Biol. 2021;220(1):e202005051. 33284322 10.1083/jcb.202005051 PMC7721913

[61] Lim YW, James D, Huang J, Lee M. The emerging role of the RNA-Binding protein SFPQ in neuronal function and neurodegeneration. Int J Mol Sci. 2020;21(19):7151. 32998269 10.3390/ijms21197151 PMC7582472

[62] Huang J, Ringuet M, Whitten AE, Caria S, Lim YW, Badhan R, Anggono V, Lee M. Structural basis of the zinc-induced cytoplasmic aggregation of the RNA-binding protein SFPQ. Nucleic Acids Res. 2020;48(6):3356–65. 32034402 10.1093/nar/gkaa076 PMC7102971

[63] Thomas-Jinu S, Gordon PM, Fielding T, Taylor R, Smith BN, Snowden V, Blanc E, Vance C, Topp S, Wong CH, et al. Non-nuclear pool of splicing factor SFPQ regulates axonal transcripts required for normal motor development. Neuron. 2017;94(4):931. 28521142 10.1016/j.neuron.2017.04.036 PMC5441113

[64] Fong JY, Pignata L, Goy PA, Kawabata KC, Lee SC, Koh CM, Musiani D, Massignani E, Kotini AG, Penson A, et al. Therapeutic targeting of RNA splicing catalysis through Inhibition of protein arginine methylation. Cancer Cell. 2019;36(2):194–209. 31408619 10.1016/j.ccell.2019.07.003 PMC7194031

[65] Zhu Y, Xia T, Chen DQ, Xiong X, Shi L, Zuo Y, Xiao H, Liu L. Promising role of protein arginine methyltransferases in overcoming anti-cancer drug resistance. Drug Resist Updat. 2024;72:101016. 37980859 10.1016/j.drup.2023.101016

[66] Parrinello S, Noon LA, Harrisingh MC, Wingfield Digby P, Rosenberg LH, Cremona CA, Echave P, Flanagan AM, Parada LF, Lloyd AC. NF1 loss disrupts Schwann cell-axonal interactions: a novel role for semaphorin 4F. Genes Dev. 2008;22(23):3335–48. 19056885 10.1101/gad.490608 PMC2600763

[67] Huang-Hobbs E, Cheng YT, Ko Y, Luna-Figueroa E, Lozzi B, Taylor KR, McDonald M, He P, Chen HC, Yang Y, et al. Remote neuronal activity drives glioma progression through SEMA4F. Nature. 2023;619(7971):844–50. 37380778 10.1038/s41586-023-06267-2 PMC10840127

[68] Smith V, Foster J, High-Risk. Neuroblastoma Treat Rev Child (Basel). 2018;5(9):114. 10.3390/children5090114 PMC6162495 30154341

[69] Del Bufalo F, De Angelis B, Caruana I, Del Baldo G, De Ioris MA, Serra A, Mastronuzzi A, Cefalo MG, Pagliara D, Amicucci M, et al. GD2-CART01 for relapsed or refractory High-Risk neuroblastoma. N Engl J Med. 2023;388(14):1284–95. 37018492 10.1056/NEJMoa2210859

[70] Killock D. CAR T cells induce durable remission of neuroblastoma. Nat Rev Clin Oncol. 2023;20(6):354. 37081160 10.1038/s41571-023-00768-9

