# Supplementary material for: Targeting TRIM59 impairs RNA splicing and promotes neuroblastoma differentiation and therapeutic responses
Source: J Exp Clin Cancer Res. 2025 Dec 18;45:25. doi: 10.1186/s13046-025-03573-7 (PMC12853711; doi:10.1186/s13046-025-03573-7)

Figure 2A

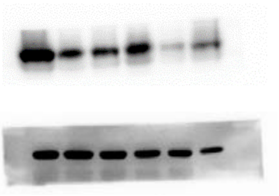

Figure 3E

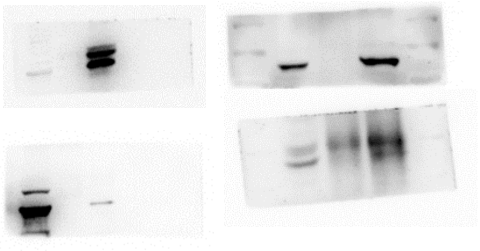

Figure 3H

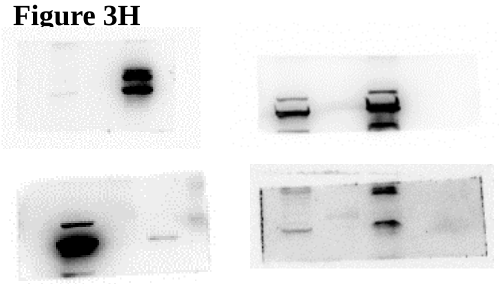

Figure 3I

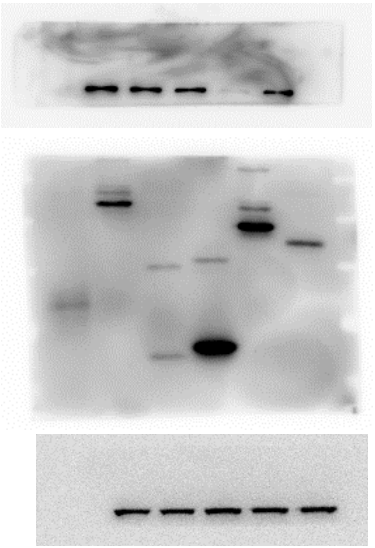

Figure 3J

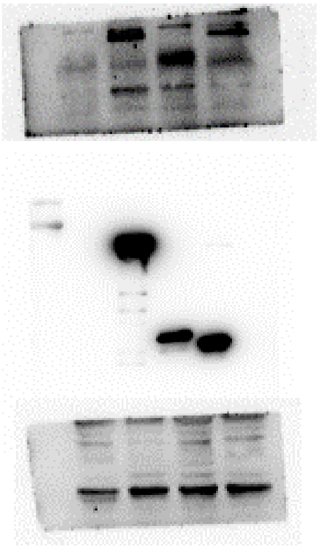

Figure 4D

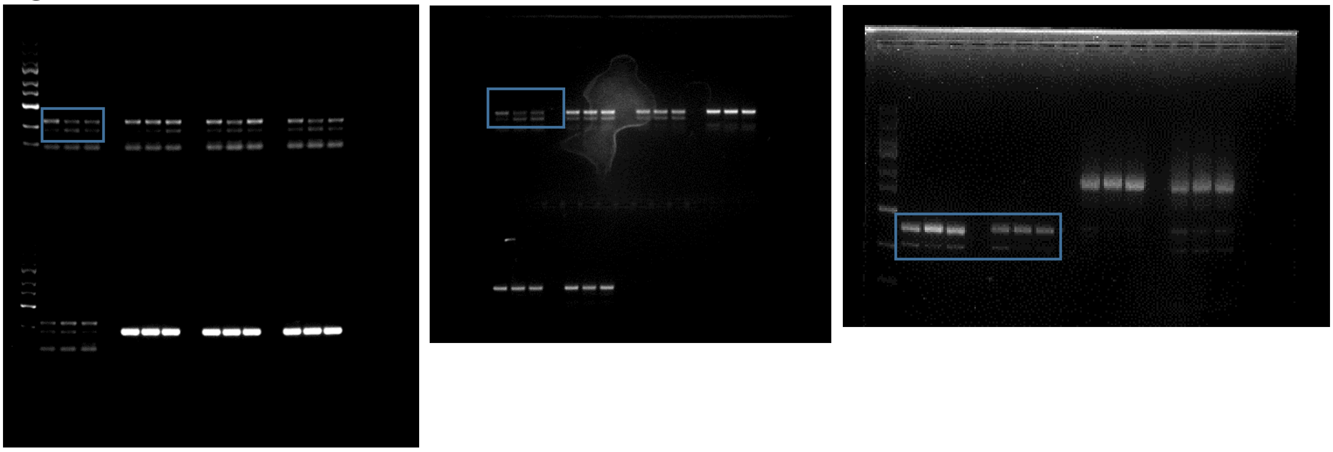

Figure 4F

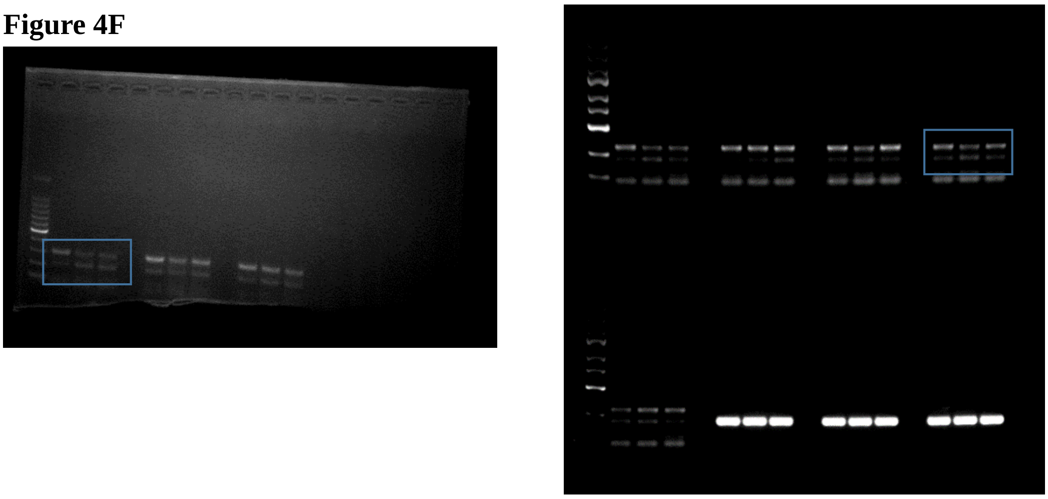

Figure 5A

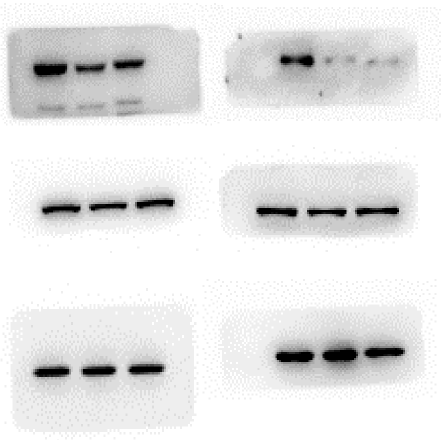

Figure 5D

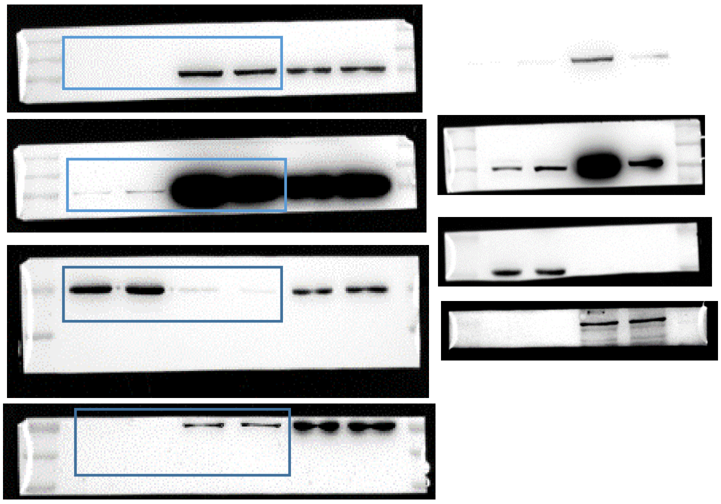

Figure 5E

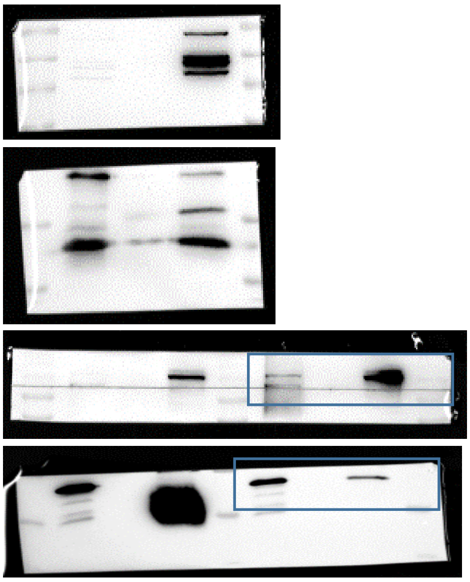

Figure 5F

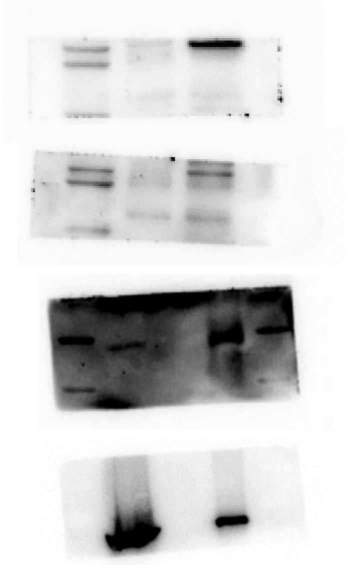

Figure 5G

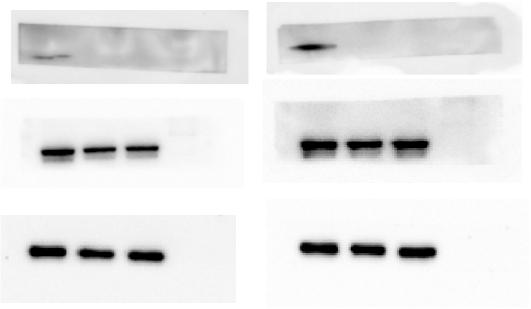

Figure 5I

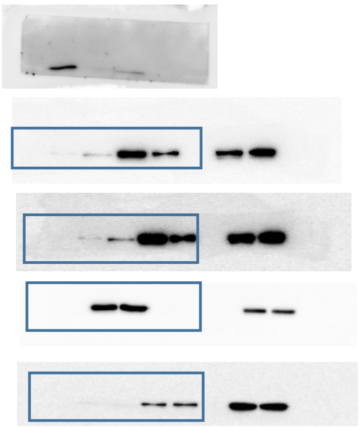

Figure 5K

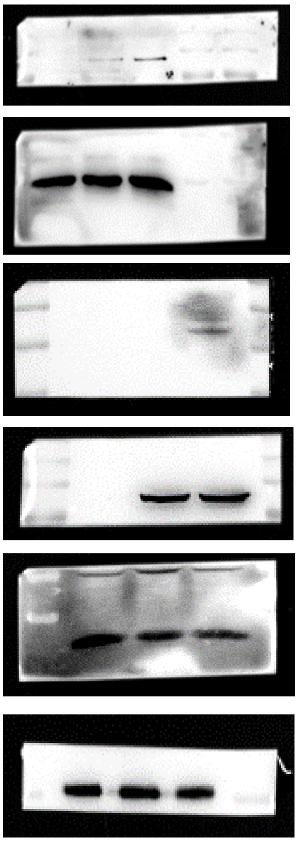

Figure 5L

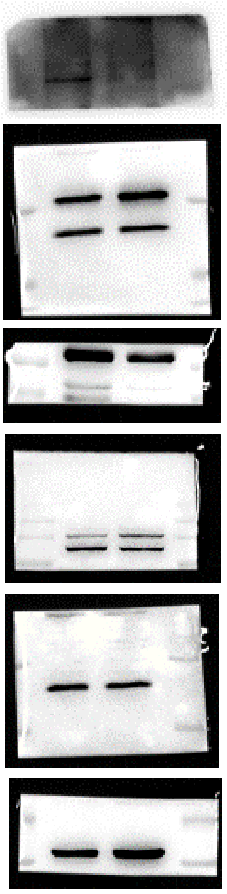

Figure 6B

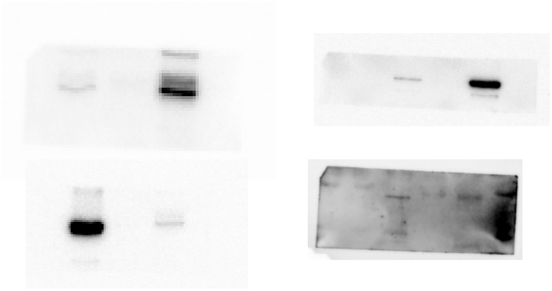

Figure 6C

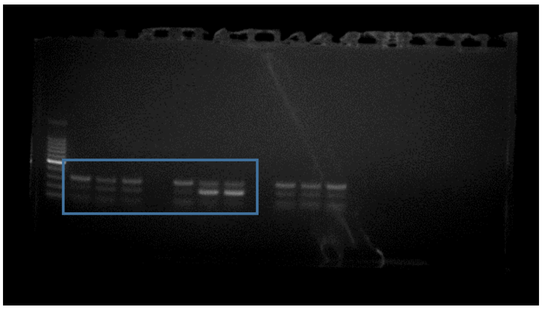

Figure 6E

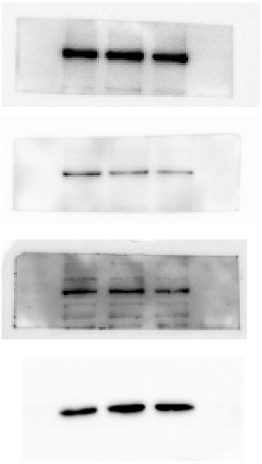

Figure 6G

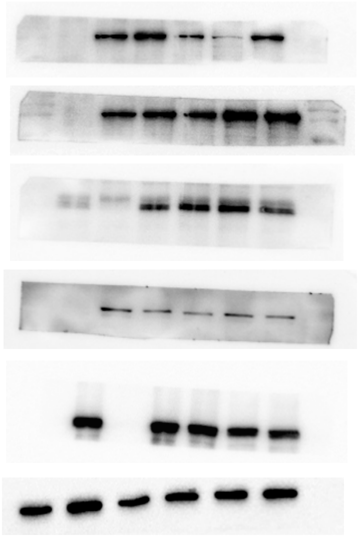

Figure 6H

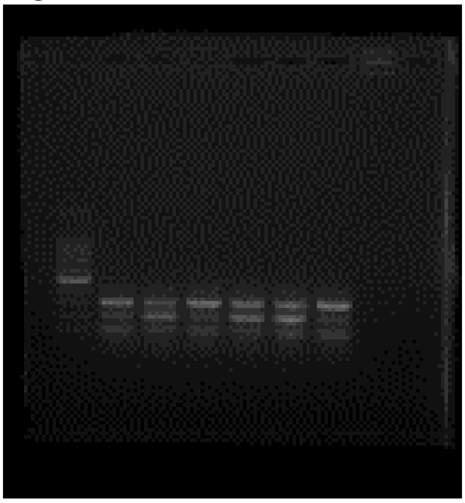

Figure 6K

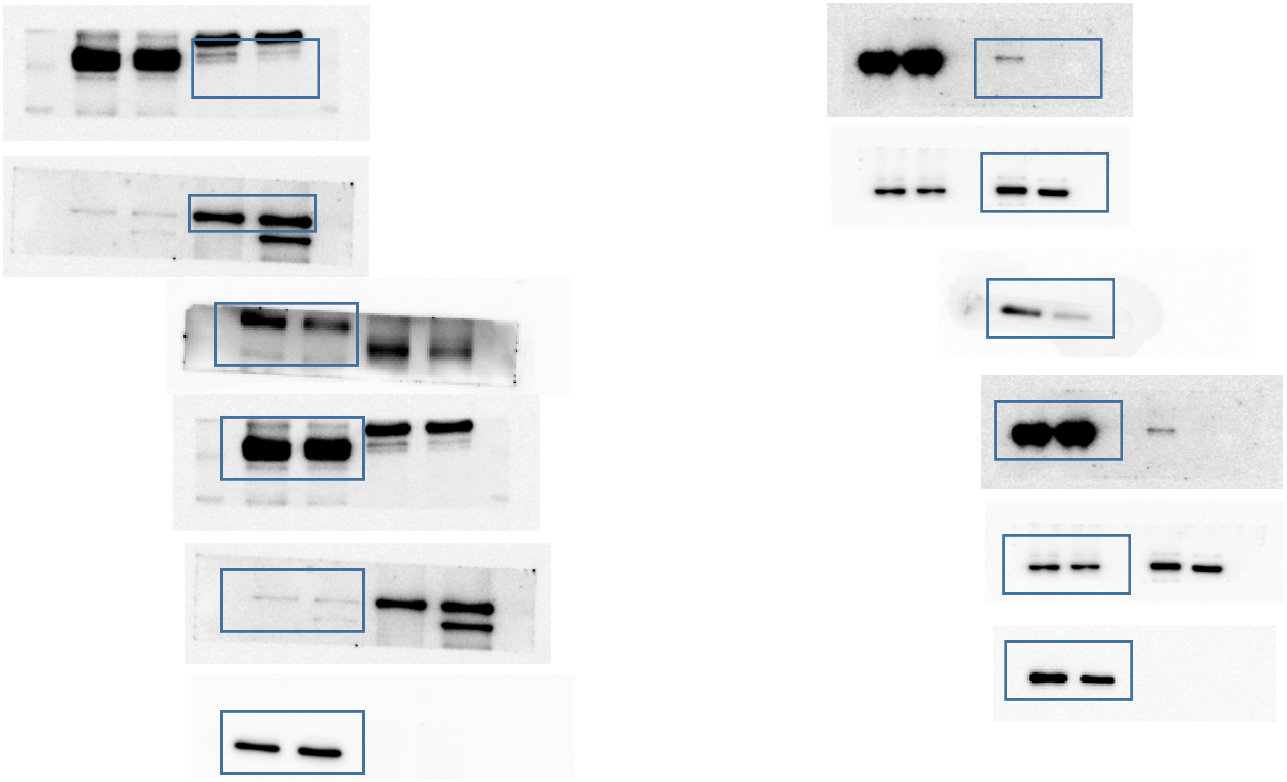

Figure 6L

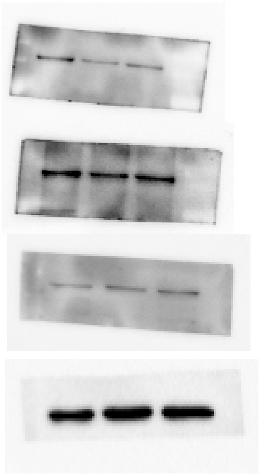

Figure 7A

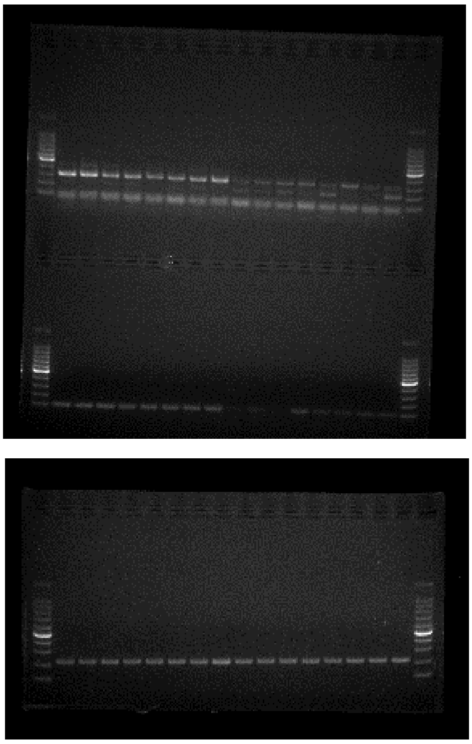

Figure 7D

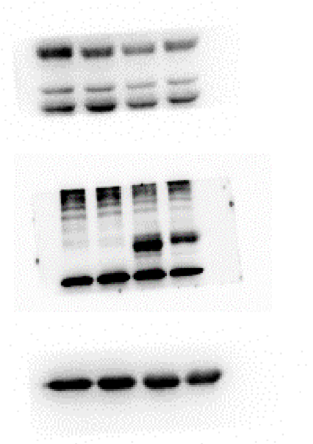

Supplemental Figure 1F

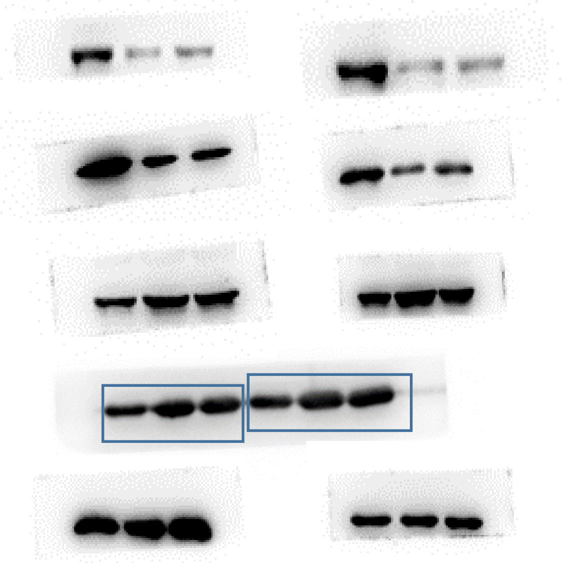

Supplemental Figure 2B

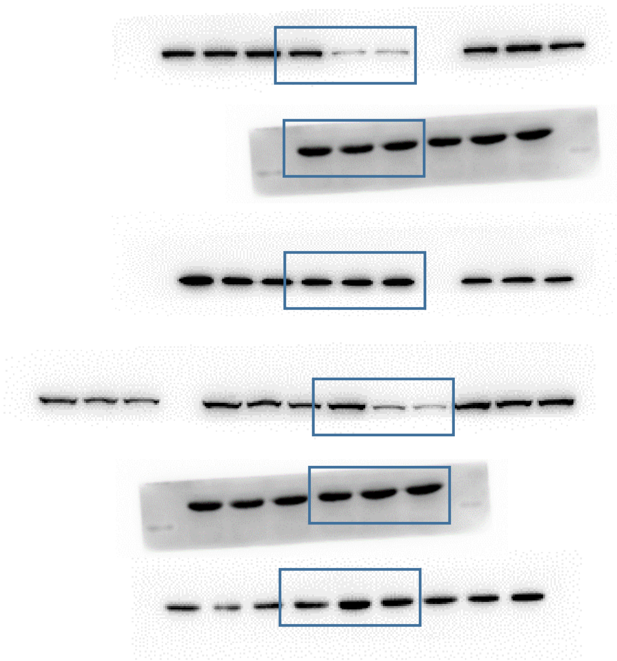

Supplemental Figure 2G

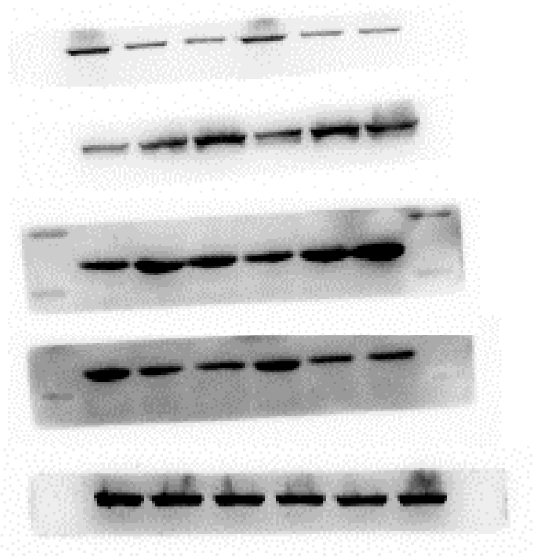

Supplemental Figure 4A

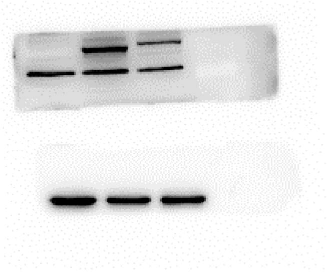

Supplemental Figure 5D

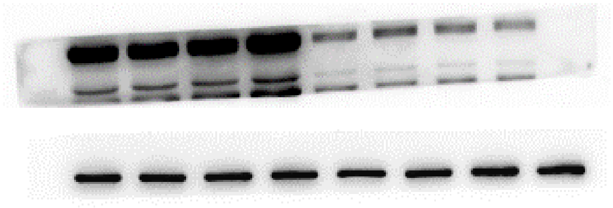

Supplemental Figure 6E

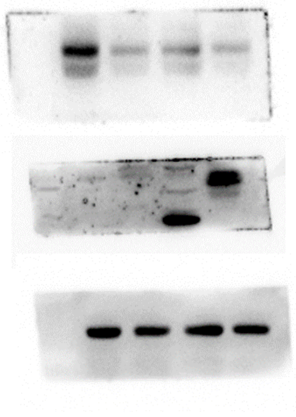

Supplement: Supplementary file 2 — Supplementary Material 2. [file 13046_2025_3573_MOESM2_ESM.pdf]
