# Supplementary material for: Targeting TRIM59 impairs RNA splicing and promotes neuroblastoma differentiation and therapeutic responses
Source: J Exp Clin Cancer Res. 2025 Dec 18;45:25. doi: 10.1186/s13046-025-03573-7 (PMC12853711; doi:10.1186/s13046-025-03573-7)
Supplement: Supplementary file 3 — Supplementary Material 3. [file 13046_2025_3573_MOESM3_ESM.docx]

Supplementary Information

**Targeting TRIM59 impairs RNA splicing and promotes neuroblastoma differentiation and therapeutic responses**

Yingwen Zhang, Yi Yang, Guoyu Chen, Minzhi Yin, Yijin Gao, Yanxin Li, and Haizhong Feng

**Correspondence**: Haizhong Feng, Email: [fenghaizhong@sjtu.edu.cn](mailto:fenghaizhong@sjtu.edu.cn)

**This Word file includes:**

**Supplemental Figure 1-6**

**
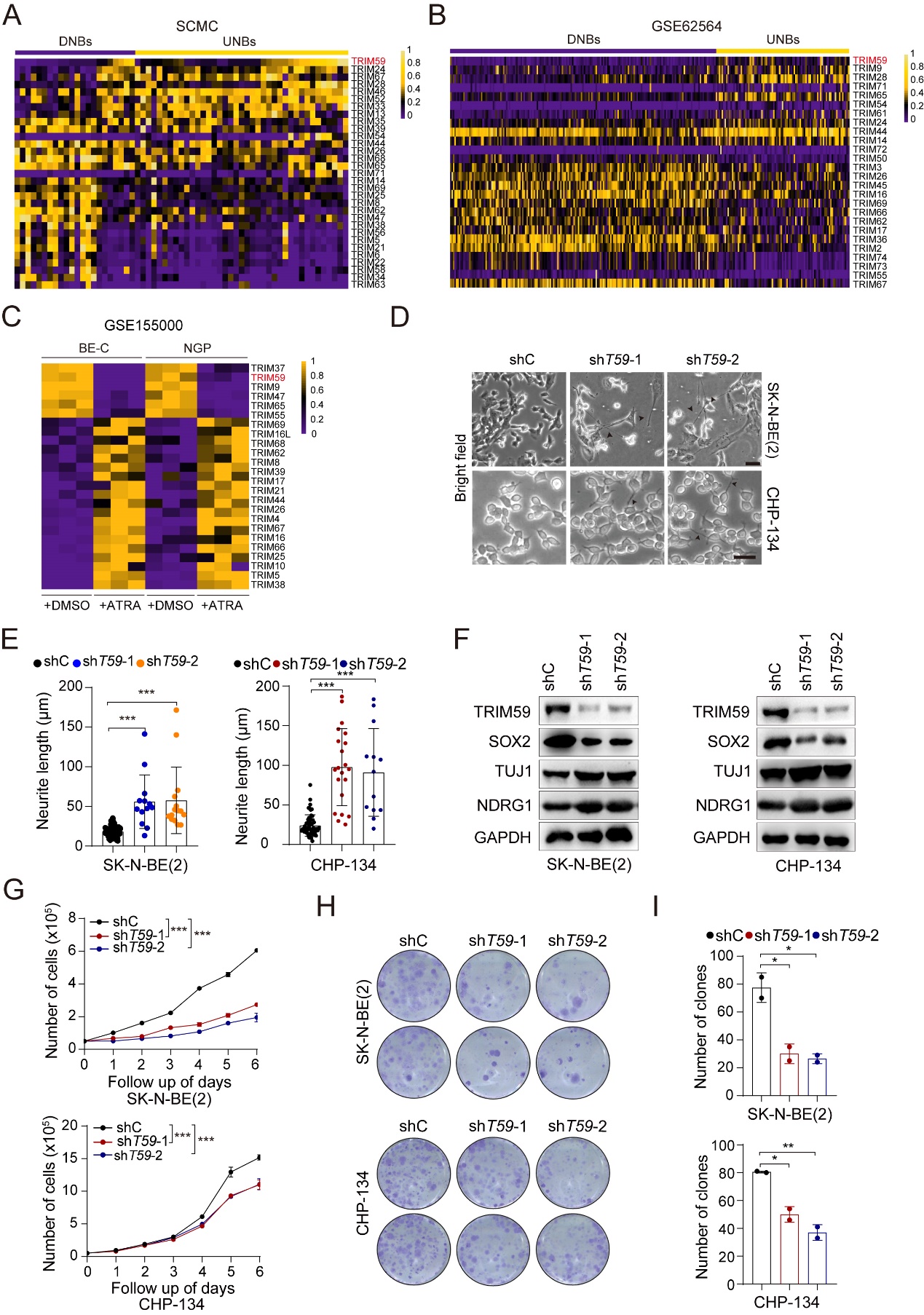
**

**Supplemental Figure 1. *TRIM59* knockdown facilitates neuroblastoma differentiation.**

1. Heatmap illustrating DEGs from TRIM family in undifferentiated (n=39) and differentiated NBs (n=22) obtained at initial diagnosis from SCMC dataset. *P*< 0.05.
2. Heatmap illustrating DEGs from TRIM family in undifferentiated (n=181) and differentiated NBs (n=91) obtained at initial diagnosis from the GSE62564 dataset. *P* < 0.05.
3. Heatmap illustrating DEGs from TRIM family in untreated and all-trans-retinoic acid (ATRA)-treated NB cell lines from the GSE155000 dataset. *P* < 0.05.
4. Representative bright-field images of SK-N-BE(2) and CHP-134 cells with shC or sh*TRIM59*. Scale bar, 50 µm.
5. Quantitative analysis of neurite lengths in SK-N-BE(2) and CHP-134 cells with shC or sh*TRIM59*.
6. Protein expression levels of SOX2, TUJ1 and NDRG1 in SK-N-BE(2) and CHP-134 cells with shC or sh*TRIM59*.
7. Cell proliferation assays.
8. Colony formation assays.
9. Quantification of survival colonies from Supplemental Figure 1H.

Data represent three independent experiments. Error bars indicate mean ± s.d. * *P* < 0.05, ** *P* < 0.01, *** *P* < 0.001, by two-tailed *t*-test.


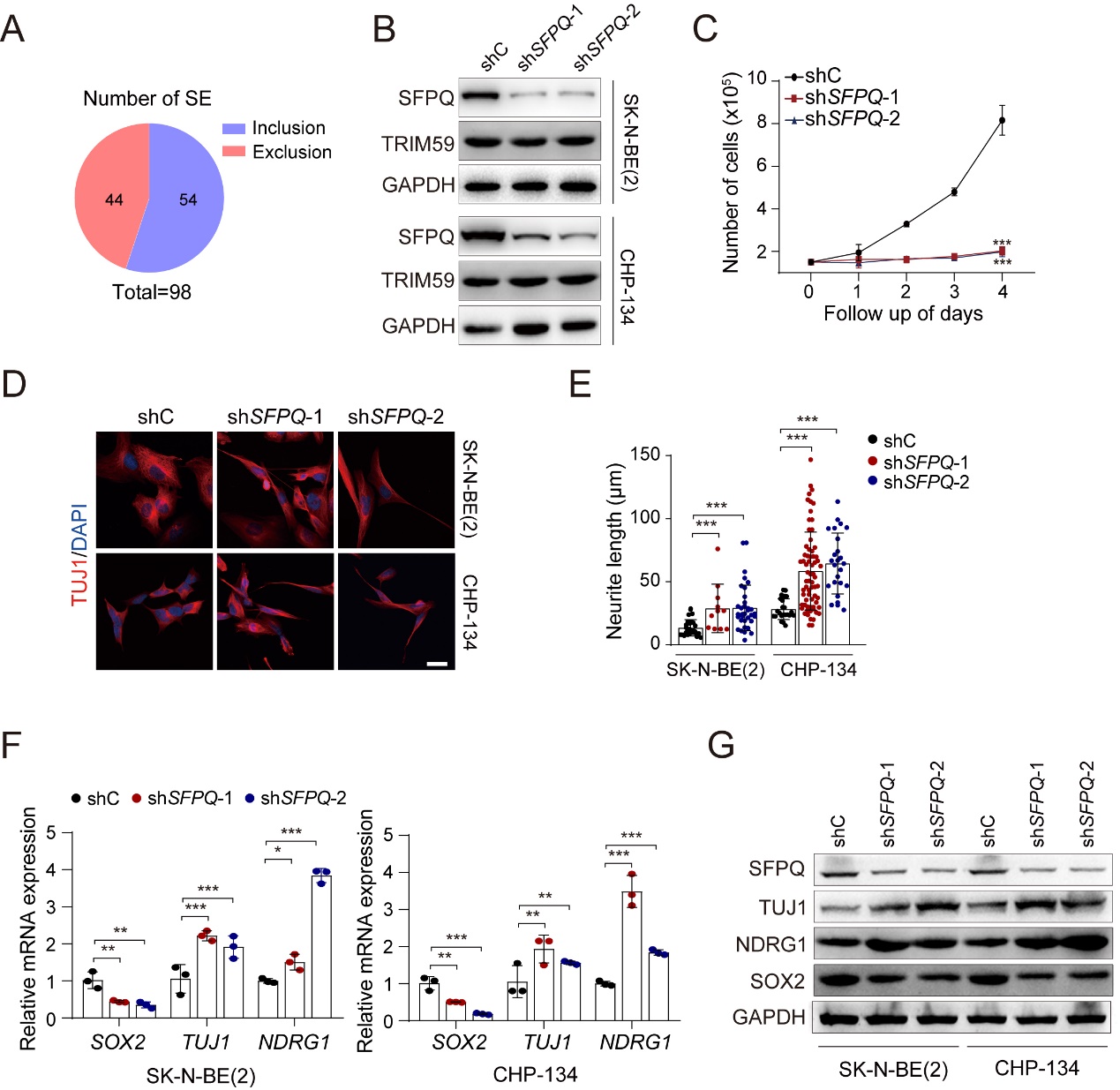


**Supplemental Figure 2. SFPQ depletion promotes neuroblastoma differentiation.**

1. Quantification of SE events affected by *TRIM59* KD.
2. WB of SK-N-BE(2) and CHP-134 cells with shC or sh*SFPQ*.
3. Cell proliferation assays.
4. IF of TUJ1 (red) and DAPI (blue).
5. Quantitative analysis of neurite lengths.
6. Relative mRNA expression levels of *SOX2*, *TUJ1*, and *NDRG1*.
7. Protein expression levels of SOX2, TUJ1 and NDRG1

Data represent three independent experiments. Error bars indicate mean ± s.d. * *P* < 0.05, ** *P* < 0.01, *** *P* < 0.001, by two-tailed *t*-test.


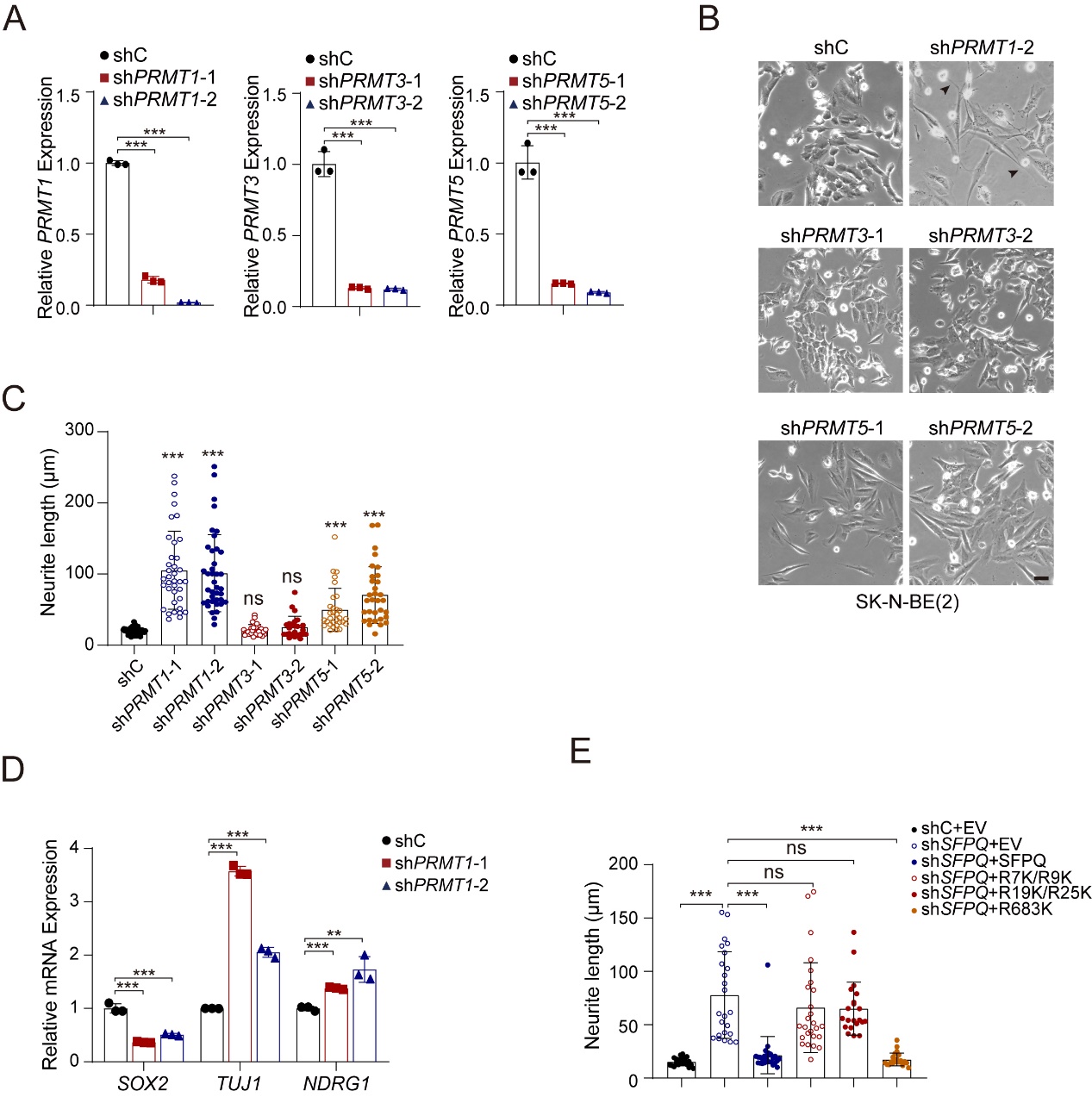


**Supplemental Figure 3. *PRMT1* depletion promotes the expression of differentiation markers.**

1. qRT-PCR of SK-N-BE(2) cells stably expressing shC, sh*PRMT1*, sh*PRMT3* or sh*PRMT5*.
2. Representative bright-field images of SK-N-BE(2) cells with shC, sh*PRMT1*, sh*PRMT3* or sh*PRMT5*. Scale bar, 50 µm.
3. Quantitative analysis of neurite lengths.
4. Relative mRNA expression levels of *SOX2*, *TUJ1*, and *NDRG1* in SK-N-BE(2) cells stably expressing shC or sh*PRMT1*.
5. Quantitative analysis of neurite lengths.

Data represent three independent experiments. Error bars indicate mean ± s.d. ns, not significant. ** *P* < 0.01, *** *P* < 0.001, by two-tailed *t*-test.

**
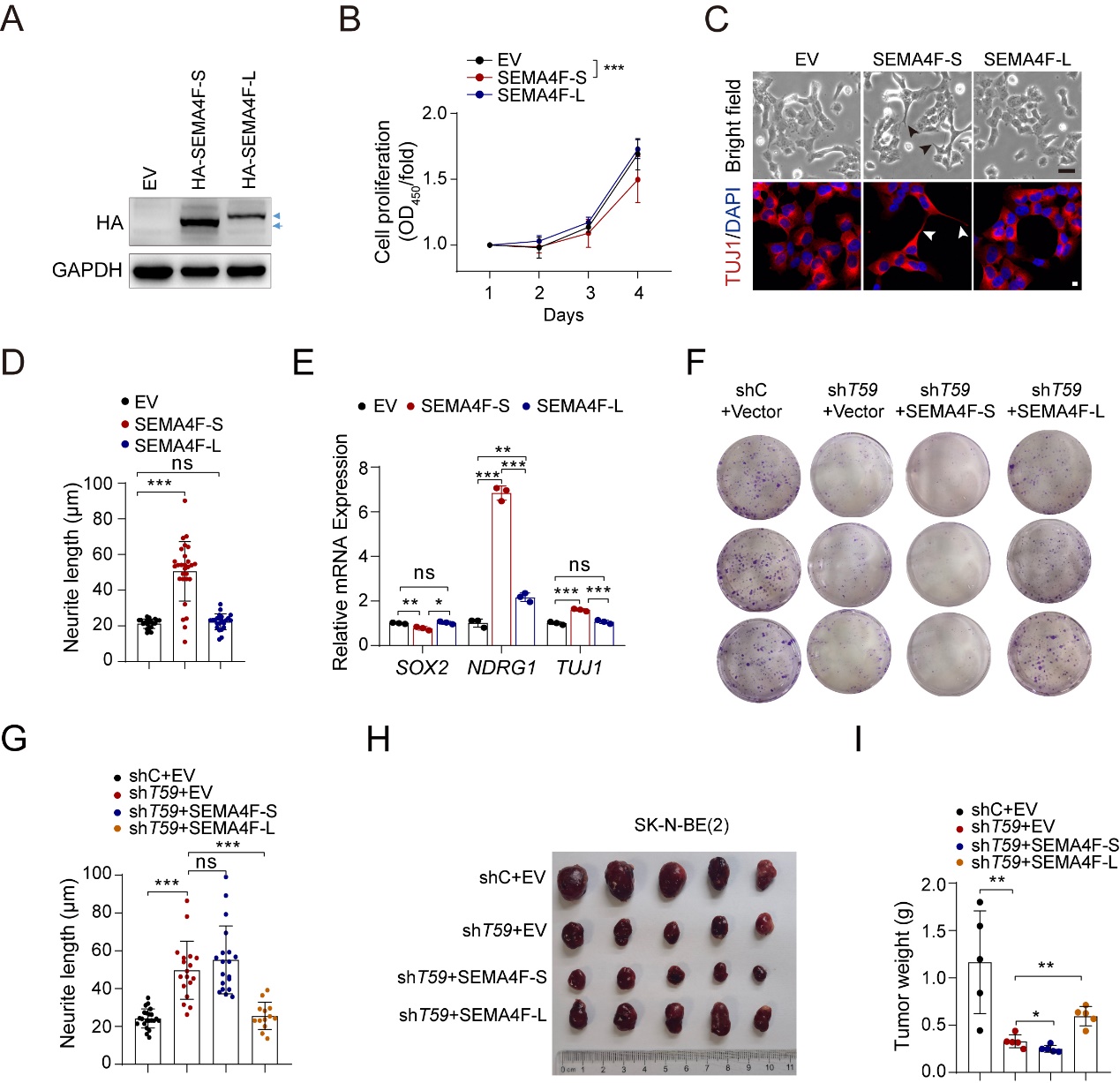
**

**Supplemental Figure 4. SEMA4F-S isoform inhibits cell proliferation and promotes NB differentiation.**

1. WB of the expression of HA-tagged SEMA4F-S and HA-tagged SEMA4F-L in SK-N-BE(2) cells. EV, empty vector. Arrows, target proteins.
2. Cell proliferation assays.
3. Representative bright-field images and TUJ1 IF analysis. Top panel: Scale bar, 50 µm. Bottom panel: Scale bar, 10 µm.
4. Quantitative analysis of neurite lengths.
5. Relative mRNA expression levels of *SOX2*, *TUJ1*, and *NDRG1*.
6. Colony formation assays.
7. Quantitative analysis of neurite lengths.
8. Representative images of tumors from 6-week-old CB17-SCID mice injected with SK-N-BE(2) cells stably expressing shC+EV, sh*TRIM59*+EV, sh*TRIM59*+SEMA4F-S or sh*TRIM59*+SEMA4F-L.
9. Quantification of tumor weight from (F).

Data represent three independent experiments. Error bars indicate mean ± s.d. * *P* < 0.05, ** *P* < 0.01, *** *P* < 0.001, ns not significant, by two-tailed *t*-test.


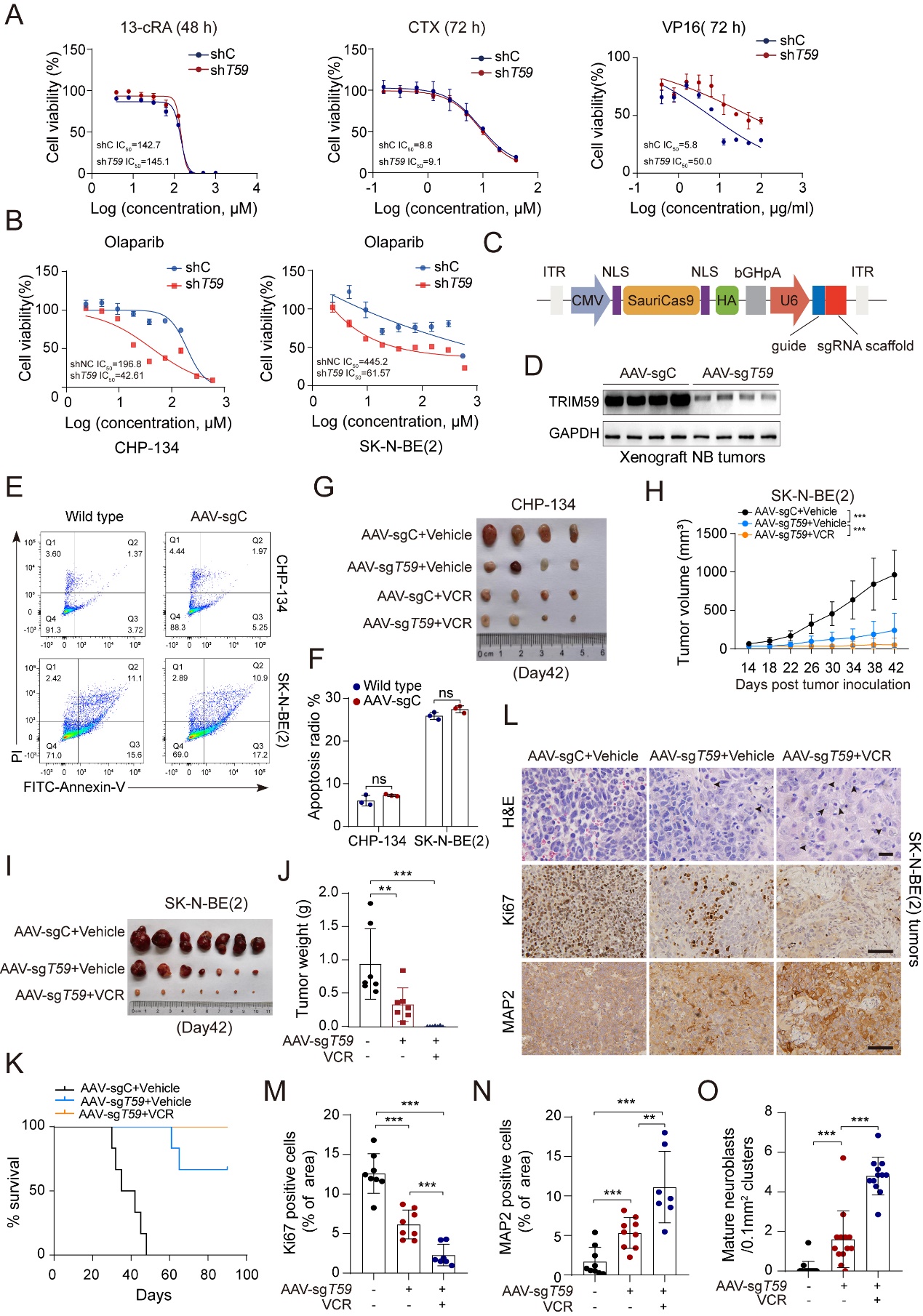


**Supplemental Figure S5. The effects of *TRIM59* knockdown on the sensitivity of NB to chemotherapy drugs.**

1. Dose-response curves of wild-type (WT) and *TRIM59* KD NB cells in response to 13-cRA, CTX, or VP16.
2. Dose-response curves of WT and *TRIM59* KD NB cells in response to olaparib.
3. Schematics of a pAAV-CMV-SauriCas9-*TRIM59*-sgRNA plasmid.
4. WB of the knockdown efficiency of TRIM59 in the xenograft model.
5. Apoptosis analysis by flow cytometry. Wild-type NB cells and control AAV–transduced NB cells were stained with Annexin V–FITC/PI and analyzed by flow cytometry.
6. Quantification of apoptosis cells.
7. Images of subcutaneous xenograft tumors derived from CHP-134 cells, one week after treatment with AVV virus and VCR (at day 42 post-transplantation).
8. Tumor growth curves recorded in SK-N-BE(2) xenograft animals.
9. Images of subcutaneous xenograft tumors derived from SK-N-BE(2) cells, one week after treatment with AVV and VCR (at day 42 post-transplantation).
10. Quantification of tumor weight from (G).
11. Kaplan-Meier survival curves of tumor xenograft studies. The subcutaneous tumor was allowed to grow to a maximum volume of 1500 mm^3^. *p* < 0.001, by the log-rank test.
12. H&E analysis, along with Ki67 and MAP2 IHC staining, of tumor sections from the CHP-134 tumors shown in (E). Top panel: Scale bar, 20 µm. Middle and bottom panel: Scale bar, 50 µm.

**(M-O)** Quantification of Ki67-positive cells **(M)**, MAP2-positive cells **(N)**, and mature neuroblasts **(O)**.

Error bars indicate mean ± s.d. * *P* < 0.05, ** *P* < 0.01, *** *P* < 0.001, by two-tailed *t*-test.

**
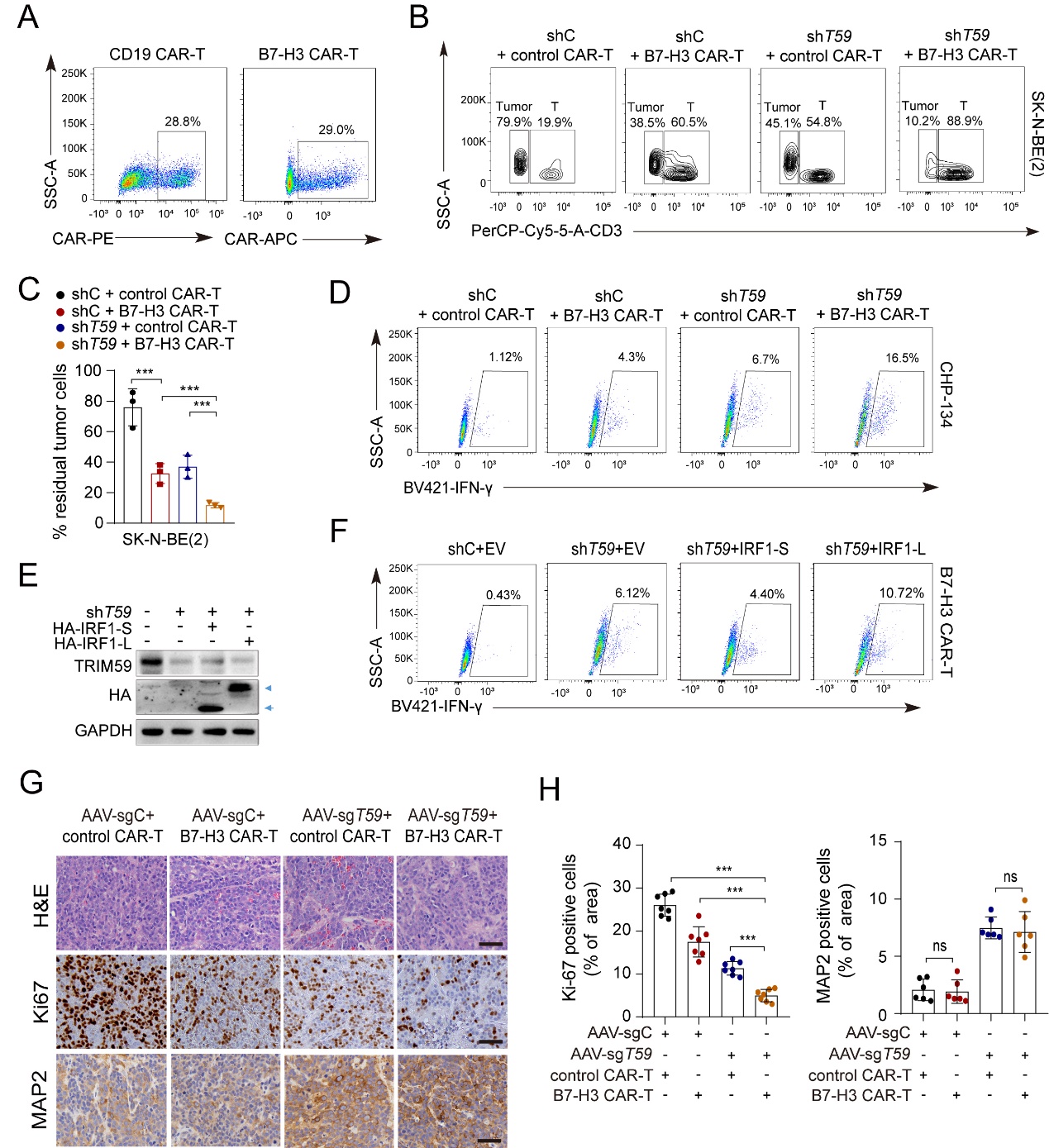
**

**Supplemental Figure S6. Depletion of TRIM59 enhances CAR-T therapy efficacy in NBs.**

1. Flow cytometry of the expression of CD19 and B7-H3 CARs in human T cells using anti-CD19 scFv or anti-B7-H3 scFv antibodies.
2. SK-N-BE(2) cells with shC or sh*TRIM59* were co-cultured with CD19 CAR-T control or B7H3 CAR-T cells at an effector-to-target ratio of 1:3 for 120 h. Tumor cells (CD3^-^) and T cells (CD3^+^) were quantified by flow cytometry.
3. Quantification of the proportion of residual tumor cells in (B).
4. Flow cytometry of IFN-γ^+^ T cells in the co-culture of *TRIM59* KD NB cells and CAR-T cells in Figure 9D.
5. WB of the re-expression of IRF1-S and IRF1-L in CHP-134/shTRIM59 cells. Arrow, target protein.
6. Flow cytometry of IFN-γ^+^ T cells in the co-culture of *IRF1* isoform-expressing NB cells and CAR-T cells in Figure 9G.
7. H&E analysis, along with Ki67 and MAP2 IHC staining, of tumor sections from the tumors shown in Figure 8K. Scale bar, 50 µm.
8. Quantification of Ki67- and MAP2- positive cells.

Error bars indicate mean ± s.d. *** *P* < 0.001, ns not significant, by two-tailed *t*-test.
